# Efficacy and Safety of TCMI in Patients With Combined Coronary Heart Disease and Heart Failure: A Systematic Review and Network Meta-Analysis 

**DOI:** 10.3389/fphar.2021.741261

**Published:** 2021-11-23

**Authors:** Penglu Wei, Kuo Yang, Dehuai Long, Yupei Tan, Wenlong Xing, Xiang Li, Hongli Wu, Hongxu Liu

**Affiliations:** ^1^ Beijing Hospital of Traditional Chinese Medicine, Capital Medical University, Beijing, China; ^2^ Institute of Medical Intelligence, School of Computer and Information Technology, Beijing Jiaotong University, Beijing, China; ^3^ Beijing University of Chinese Medicine, Beijing, China; ^4^ Institute of Basic Research in Clinical Medicine, China Academy of Chinese Medical Sciences, Beijing, China

**Keywords:** traditional Chinese medicine injection, coronary heart disease, heart failure, network meta-analysis, systematic review

## Abstract

**Objective:** To compare the efficacy and safety of conventional treatments (CTs) to those that included traditional Chinese medicine injections (TCMIs) in patients with combined coronary heart disease and heart failure (CHD-HF).

**Methods:** Eight electronic literature databases (PubMed, Embase, Cochrane Central Register of Controlled Trials, Web of Science, China National Knowledge Infrastructure Database, Chinese Scientific Journal Database, Wanfang Database, Chinese Biomedical Database) were searched from their inceptions to May 18, 2021, to identify relevant randomised controlled trials (RCTs). The primary outcomes analyzed included the total effectiveness rate and adverse events (ADRs). The secondary outcomes analyzed included the left ventricular ejection fraction (LVEF), N-terminal pro-brain natriuretic peptide (NT-proBNP), brain natriuretic peptide (BNP), and 6-min walk test (6MWT). Cochrane risk-of-bias tool was used to assess quality of the analyzed RCTs. Stata and OpenBUGS software were used to prior to the systematic review and network meta-analysis.

**Results:** Sixty-one eligible trials involved 5,567 patients and one of the following 15 TCMIs: Shuxuetong, Shenmai, Shenfu, Shengmai, Danshenduofenyansuan, Danhong, Dazhuhongjingtian, Xinmailong, Dengzhanxixin, Gualoupi, Shuxuening, Xuesaitong, Yiqi Fumai, Shenqi Fuzheng, Huangqi. Network meta-analysis revealed that Shuxuetong injection + CT group was superior to CT only in improving the total effectiveness rate [odds ratio (OR): 7.8, 95% confidence interval (CI): 1.17–27.41]. Shenmai injection + CT was superior to CT only for LVEF (OR: 8.97, CI: 4.67–13.18), Xinmailong injection + CT was superior to CT only for NT-proBNP (OR: −317.70, CI: −331.10–303.10), Shenqi Fuzheng injection + CT was superior to CT only for BNP (OR: −257.30, CI: −308.40–242.80); and Danhong injection + CT was superior to CT only for 6MWT (OR: 84.40, CI: 62.62−106.20). Different TCMIs had different toxicity spectrums.

**Conclusion:** TCMIs combined with CT are better than CT alone in treating CHD-HF. Different TCMIs improve different outcomes. Additional properly designed RCTs are needed to conduce a more refined comparison of various TCMIs.

**Systematic Review Registration:** [https://www.crd.york.ac.uk/PROSPERO/], identifier [CRD42021258263].

## Introduction

Heart failure (HF) is a heterogeneous clinical syndrome and represents the final path of various heart diseases (Pagliaro et al., 2019), with an estimated 64.3 million people suffering from HF worldwide (GBD 2017 Disease and Injury Incidence and Prevalence Collaborators, 2018). The latest epidemiological survey of HF in China shows that its prevalence rate among residents over 35 years old is 1.3%; accordingly, it is estimated that there are about 8.9 million HF patients (Hao et al., 2019; Metra and Lucioli, 2020). Ischemic heart disease is one of the most frequent causes of HF. It is usually attributed to coronary heart disease (CHD), which is defined by the presence of one or more obstructive plaques that lead to reduced coronary blood flow, myocardial ischemia, and subsequent HF (Lala and Desai, 2014; Cleland and Pellicori, 2019; Severino et al., 2020).

HF and CHD share many risk factors. Cardiovascular risk factors such as hypertension and diabetes promote atherosclerosis development, leading to CHD. HF can result from CHD or other specific cardiovascular risk factors (Taylor and Hobbs, 2013). Conventional treatments of CHD-HF include diuretics, angiotensin converting enzyme inhibitors (ACEIs), angiotensin II receptor blockers (ARBs), β-receptor blockers, anti-platelet and anti-thrombotic drugs, statins, aldosterone-receptor blockers, digoxin, and vasodilator agents (Committee of Exports on Rational Drug Use National Health and Family Planning Commission of the People’ Republic of China, Chinese Pharmacists Association, 2019; Elgendy et al., 2019; Lee et al., 2019). However, these treatments have many adverse effects, such as hypotension, arrhythmias, neuropsychosis, hyperkalemia, and worsening kidney function, which limit their clinical applications (Moser, 1997; Saedder et al., 2014). Although non-pharmacological treatments such as coronary artery bypass graft surgery (CABG), percutaneous transluminal angioplasty (PTCA), cardiac resynchronization therapy (CRT), and heart transplantation have been used in the treatment of CHD-HF, a significant number of CHD-HF patients still have no access to effective treatments (Sardu et al., 2017). Hence, it is important to explore other potentially effective interventions for treating CHD-HF.

Traditional Chinese medicine injections (TCMIs) have been widely used to treat CHD-HF (Hu et al., 2009; Zhu and Han, 2014; Xian et al., 2016; Xu and Cao, 2019). Dozens of RCTs and pairwise meta-analyses using direct comparison models have been carried out to compare the efficacy and safety of TCMIs in patients with CHD-HF (Jiang and Shang, 2018; Wei et al., 2020). Since no head-to-head RCTs comparisons involving TCMIs are available, indirect comparisons involving networks of studies linked by one or more common comparators can be used to assess the efficacy and safety of different TCMIs in patients with CHD-HF (Bucher et al., 1997; Cooper et al., 2019). Network meta-analysis can synthesize evidence from direct and indirect comparisons to identify the best available treatment (Cipriani et al., 2013). Here, we described our network meta-analysis of relevant RCTs conducted with the goal to evaluate the relative efficacy and safety of different TCMIs in patients with CHD-HF.

## Methods

This study was conducted following the protocol registered with PROSPERO (Protocol number: CRD42021258263). Our network meta-analysis was performed in accordance with the Preferred Reporting Items for Systematic Reviews and Meta-Analyses (PRISMA) guidelines (Radua, 2021; Hutton et al., 2015); see Supplementary Table S1. Bayesian network meta-analysis was applied to make probabilistic statements and predictions regarding treatment effects and advantages in complex clinical situations (Salanti et al., 2011).

### Data Sources and Searches

We searched PubMed, Embase, Cochrane Central Register of Controlled Trials, Web of Science, China National Knowledge Infrastructure Database, Chinese Scientific Journal Database, Wanfang Database, and Chinese Biomedical Database to get relevant articles with no language restrictions published before May 18, 2021, using as the main search term (“Coronary Diseases”) or (“Heart failure”) and (“Injection”) within the restriction limit of (“randomized controlled trial”). A subset of Chinese and English journals that might publish studies relevant for our subject were also searched manually. The detailed search strategy is described in Supplementary Table S2.

### Study Selection

Two review authors (PW and KY) independently reviewed the titles and abstracts of trials retrieved by the search for potential eligibility. Then, we acquired the full texts of trials considered potentially eligible for inclusion in the review. We sought further information from the authors of the trial, which was not sufficient to determine eligibility. Any differences were resolved through consensus and arbitration by a panel of adjudicators (PW, DL, YT, WX, and XL).

We included published RCTs that met the following criteria:

• Participants: all the enrolled participants were required to accord with the current or past definitions of CHD and HF (Hu et al., 2009; Zhu and Han, 2014; Xian et al., 2016; Xu and Cao, 2019; Pan et al., 2005; Yuan and Du, 2012; Feng, 2013; Wang et al., 2019; Li et al., 2016a; Shi et al., 2016; Shen et al., 2017; Ji, 2019; Zhou et al., 2005; Wu and Duan, 2009; Yang, 2009; Zhao et al., 2011a; Cao, 2012; Dong, 2012; Shen, 2012; Wu and Huang, 2012; Yang and Li, 2012; Zhou et al., 2013; Luo et al., 2015; Xiu and Chen, 2015; Li et al., 2016b; He, 2016; Mao, 2016; Wang et al., 2016; Wu, 2016; Li et al., 2018; Wang and Jang, 2018; Li, 2019a; Zhou and Luo, 2020; Lu, 2005; Zhu et al., 2008; Xing et al., 2009; Wang, 2012; Zhao et al., 2012; Wu, 2014; Yang et al., 2014; Zhang, 2015a; Teng, 2016; Xu, 2016; Tian et al., 2017; Zhan et al., 2017; Han, 2018; Ni et al., 2020; Zhang, 2020; Huang et al., 1999; Zhao et al., 2011b; Wang et al., 2011; Zhang, 2015b; Wei and Lu, 2020; Ren, 2021; Guo et al., 2012). Trials without a description of the detailed diagnostic criteria but which reported patients with definite CHD-HF were also included (Zhou et al., 2002; Kuang, 2004; Xin and Shan, 2012; Wu et al., 2017; Gong et al., 2018; Li, 2019b).
• Interventions: the control group was treated with a conventional treatment only, including diuretics, ACEIs, or ARBs, β-receptor blocker, aldosterone-receptor blocker, digoxin, or vasodilator substance, while the experimental group was treated with a conventional treatment and one of the following 15 TCMIs: Shuxuetong, Shenmai, Shenfu, Shengmai, Danshenduofenyansuan, Danhong, Dazhuhongjingtian, Xinmailong, Dengzhanxixin, Gualoupi, Shuxuening, Xuesaitong, Yiqi Fumai, Shenqi Fuzheng, Huangqi.
• Outcomes: the primary outcomes were total effectiveness rate and adverse reactions (ADRs). The secondary outcomes included the left ventricular ejection fraction (LVEF), N-terminal pro-brain natriuretic peptide (NT-proBNP), brain natriuretic peptide (BNP), and 6-min walk test (6MWT). The included trials were required to report at least one of these clinical outcome measures.

Studies not meeting all these inclusion criteria were excluded. In addition, the following exclusion criteria were applied:

• Interventions in the control group included other traditional treatments, such as other TCMI, acupuncture, or Chinese herbal medicine.
• The criteria of efficiency evaluation did not meet the following definitions (Hu et al., 2009; Xian et al., 2016; Hu et al., 2009; Xian et al., 2016; Yuan and Du, 2012; Feng, 2013; Shi et al., 2016; Shen et al., 2017; Wu et al., 2017; Wu and Huang, 2012; He, 2016; Gong et al., 2018; Wu and Huang, 2012; He, 2016; Gong et al., 2018; Dong, 2012; Li et al., 2018; Li, 2019a; Wu and Duan, 2009; Luo et al., 2015; Xiu and Chen, 2015; Wu and Duan, 2009; Luo et al., 2015; Xiu and Chen, 2015; Zhao et al., 2012; Li, 2019b; Yang et al., 2014; Zhang, 2015a; Xu, 2016; Tian et al., 2017; Zhang, 2020; Xing et al., 2009; Teng, 2016; Zhao et al., 2011b; Wang et al., 2011; Zhang, 2015b; Zhao et al., 2011b; Wang et al., 2011; Zhang, 2015b): (1) Excellent: HF was prominently ameliorated and/or the New York Heart Association functional class (NYHA) classification improved to I level or increased by at least two levels; (2) Valid: HF was partially ameliorated, or NYHA classification increased by at least one level; (3) Invalid: HF was not ameliorated or NYHA classification was unchanged between before and after treatment, or an exacerbation or death occurred. The total effectiveness rate was calculated as the sum of the marked effectiveness rate and the effectiveness rate.

### Data Extraction and Risk of Bias Assessment

Data extraction and quality assessment were independently performed by two investigators (PW and HW). Data on trial details are as follows: (1) Basic information of the eligibility, including the content of study ID, first author, nationality, publication year, and study design; (2) Basic characteristics of included patients: sample size, sex composition, average age, course of treatment, and population distribution with the NYHA class; (3) Details of interventions; (4) Details of outcomes; (5) Information of quality assessment of RCTs. Two investigators (PW and KY) independently assessed risk of bias of individual studies. Discrepancies were resolved through consensus and arbitration by a panel of adjudicators (PW, DL, YT, WX, and XL). We also made attempts to contact the study authors by means of email, phone, or fax to obtain missing demographic information, such as the sample size, sex distribution, age, etc. When studies had multiple publications, we sorted all reports of the same study, so that each study, not each report, was the unit of interest in the review, and these studies were given a single study ID.

We assessed risk of bias of included RCTs using the Cochrane Risk of Bias Tool (Zhao et al., 2011b; Wang et al., 2011; Zhang, 2015b) based on the following items: random sequence generation, allocation concealment, blinding of participants and personnel, blinding of outcome assessment, incomplete outcome data, selective reporting, and other bias. Each item was scored as low, unclear, or high risk of bias. Any disagreements were resolved by a third researcher.

### Data Synthesis and Statistical Analysis

We synthesized all direct and indirect evidence to compare different treatments in terms of efficacy and safety, reported as odds ratios for binary outcomes (total effectiveness rate and adverse events) along with the corresponding 95% confidence intervals (CIs). Using Stata (version 16.0), we generated network diagrams for different outcomes to illustrate geometries, to clarify which treatments were directly or indirectly compared in the included studies (Chaimani et al., 2013). We analyzed frequency and random effects and conducted pairwise meta-analysis for head-to-head comparisons based on two or more trials. We assessed heterogeneity between the studies using Q test and the I^2^ statistic within a visual forest plot. A *p* value less than 0.05 was regarded statistically significant. Heterogeneity was considered low, moderate, or high for estimated I^2^ values under 25%, between 25% and 50%, and over 50%, respectively (Higgins et al., 2003).

Network meta-analyses were performed in a Bayesian framework using a Markov Chain Monte Carlo simulation technique using OpenBUGS (version 3.2.3). For all outcomes (total effectiveness rate, LVEF, NT-proBNP, BNP, and 6MWT), 150,000 sample iterations were generated with 100,000 burn-ins and a thinning interval of 1. We evaluated convergence of iterations by visual inspection of the three chains to establish homogenous parameter estimates in accordance with the Brooks-Gelman-Rubin diagnostic (Supplementary Figure S1) (Zhao et al., 2011b; Wang et al., 2011; Zhang, 2015b). Within the Bayesian framework, the network meta-analysis estimates the overall ranking of treatments by calculating the surface under the cumulative ranking curve for each; it is equal to 1 when the treatment is definitely best and 0 when the treatment is definitely the worst (Salanti et al., 2011). To assess the robustness and reliability of the results, we performed sensitivity analysis. We restricted the case number ≥100 to observe the effect of various treatments in patients with HF.

## Results

### Results of the Search

**FIGURE 1:**
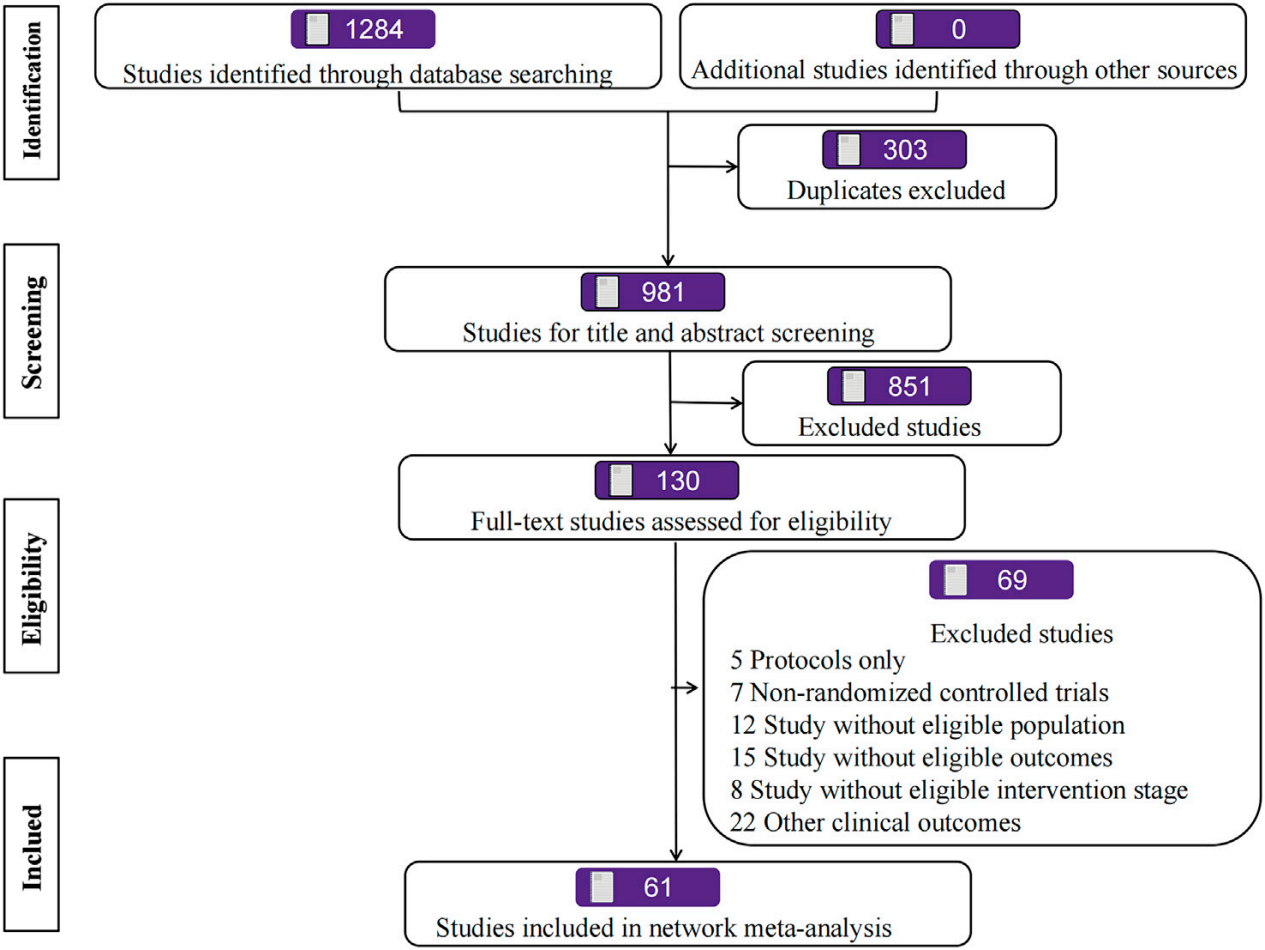
Flow chart of the study selection.

Our search strategy initially identified 1,284 records. After removal of duplicates, 981 remained for screening based on their titles and abstracts, of which 851 were excluded as irrelevant. We reviewed 130 full-text articles or, if these were not available, abstract publications or trial registry entries. Finally, we identified 61 RCTs (Hu et al., 2009; Zhu and Han, 2014; Xian et al., 2016; Xu and Cao, 2019; Hu et al., 2009; Zhu and Han, 2014; Xian et al., 2016; Xu and Cao, 2019; Hu et al., 2009; Zhu and Han, 2014; Xian et al., 2016; Xu and Cao, 2019) for inclusion, all of which had published. A study flow diagram is presented in Figure 1.

### Systematic Review and Characteristics

Among 61 RCTs (Hu et al., 2009; Zhu and Han, 2014; Xian et al., 2016; Xu and Cao, 2019; Hu et al., 2009; Zhu and Han, 2014; Xian et al., 2016; Xu and Cao, 2019; Hu et al., 2009; Zhu and Han, 2014; Xian et al., 2016; Xu and Cao, 2019) for inclusion, a total of 5,567 patients enrolled who received one of the 15 different treatments TCMIs, as listed in the Methods, in combination with conventional treatments (diuretics, ACEIs, ARBs, β-receptor blocker, aldosterone-receptor blocker, digoxin, or vasodilator substance). The main characteristics of all included studies are depicted in Table 1. The detailed information of TCMIs is described in Supplementary Table S3. Available data about absolute efficacy of various TCMIs are described in Supplementary Table S4.

**TABLE 1:** Baseline characteristics of studies included in the network meta-analysis.

Included studies	Sample size	Sex	Age	NYHA class (II-IV)	Intervention arm	Control arm	Course	Outcomes
	(E/C)	(M/F)	(E/C)	(E/C)	(E)	(C)	(days)
Hu et al. (2009)	31/32	24/39	72.94 ± 7.58/76.43 ± 4.88	0,20,11/0,22,10	Shenfu injection 40 ml ivgtt qd + CT	CT	7	②③
Xian et al. (2016)	114/114	137/91	68.95 ± 9.91/68.12 ± 8.88	55,50,9/56,43,15	Shenmai injection 100 ml ivgtt qd 20–40 drops per minute + CT	placebo + CT	7	②③⑤⑥
Xin and Shan (2012)	28/28	31/26	58.9 ± 8.7/59.6 ± 9.2	5,14,9/6,18,4	Shengmai injection 40 ml ivgtt qd + CT	CT	7	①③
Zhu and Han (2014)	50/50	59/41	66.2 ± 11.41/68.98 ± 10.28	3,47,10	Yiqi Fumai Lyophilized Injection 5.2 g ivgtt qd 20 drops per minute + CT	CT	14	①②
Feng (2013)	27/24	NR	56–86	12,12,3/10,12,2	Yiqi Fumai Lyophilized Injection 5.2 g ivgtt qd 30 drops per minute + CT	CT	14	①③⑤
Yuan and Du (2012)	82/80	120/42	45–98	NR	Yiqi Fumai injection 5.2 g ivgtt qd + CT	CT	10	②③⑥
Pan et al. (2005)	30/30	40/20	51–79	0,44,18	Shengmai injection 100 ml ivgtt qd + CT	CT	14	①
Wang et al. (2019)	74/70	43/37	68.58 ± 8.42/68.14 ± 8.73	20,12,8/17,13,10	Shenfu injection 50 ml ivgtt qd + CT	placebo + CT	7 ± 1	①②
Xu and Cao (2019)	57/51	49/59	61.39 ± 5.73/60.28 ± 6.41	19,38,0/17,34,0	Xinmailong injection 5 mg/kg ivgtt bid + CT	CT	7	①③④
Wu et al. (2017)	48/42	59/31	54.05 ± 3.96/56.13 ± 4.87	NR	Xinmailong injection 5 mg/kg ivgtt bid 20–40 drops per minute + CT	CT	10	③⑤⑥
Shi et al. (2016)	58/58	57/59	56.2 ± 8.74/55.6 ± 9.18	NR	Xinmailong injection 5 mg/kg ivgtt bid 20–40 drops per minute + CT	CT	5	③⑤
Shen et al. (2017)	58/58	70/46	62.8 ± 7.1/61.6 ± 7.8	7,42,9/8,40,10	Xinmailong injection 4 ml ivgtt bid + CT	CT	14	①③④⑥
Li et al. (2016a)	36/36	38/34	70.2 ± 2.9/71.3 ± 1.2	NR	Xinmailong injection 5 mg/kg ivgtt bid + CT	CT	5	①④
Ji (2019)	45/45	53/37	65.48 ± 5.1/65.05 ± 5.02	15,30,0/16,29,0	Xinmailong injection 4 ml ivgtt bid 20–40 drops per minute + CT	CT	14	①④
Gong et al. (2018)	45/45	56/34	68.61 ± 5.12/63.11 ± 1.45	NR	Xinmailong injection 5 mg/kg ivgtt bid + CT	CT	14	②③
Wu and Huang (2012)	45/30	38/37	70.98 ± 11.24/66.07 ± 11.74	0,25,20/0,17,13	Shenfu injection 60 ml ivgtt qd + CT	CT	14	①③
He (2016)	45/45	59/31	61.4 ± 8.3/62.3 ± 7.8	21,24,0/23,22,0	Shenfu injection 60 ml ivgtt qd + CT	CT	14	③⑤
Wang et al. (2016)	26/30	29/27	71.56 ± 2.47/70.23 ± 1.56	NR	Shenfu injection 60 ml ivgtt qd 30 ml/h + CT	CT	10 ± 2	①②④
Yang (2009)	30/30	42/18	62.8 ± 6.9	8,32,20	Shenfu injection 50 ml ivgtt qd + CT	CT	14	①②
Wu (2016)	60/60	44/76	82.5 ± 10	37,15,8/35,17,8	Shenfu injection 60 ml ivgtt qd + CT	CT	20	③
Zhou et al. (2013)	30/30	34/26	62–88/60–87	5,17,8/6/18/6	Shenfu injection 40 ml ivgtt qd + CT	CT	14	①②④
Zhou et al. (2005)	30/30	42/18	62.8 ± 6.9	8,32,20	Shenfu injection 50 ml ivgtt qd + CT	CT	14	①②
Wang and Jang (2018)	25/25	31/19	54 ± 11.1/53.6 ± 11.8	NR	Shenfu injection 40 ml ivgtt qd + CT	CT	14	①④
Zhou Luo (2020)	41/41	41/41	68.62 ± 2.47/68.7 ± 2.42	NR	Shenfu injection 50 ml ivgtt qd + CT	CT	14	②④
Dong (2012)	30/30	31/29	59.8 ± 10.2/61.7 ± 10.6	10,12,8/11,13,6	Shenfu injection 50 ml ivgtt qd + CT	CT	14	①②③⑤
Li et al. (2018)	30/30	30/20	63.8 ± 12.8/65.2 ± 11.3	25,5,0/16,14,0	Shenfu injection 60 ml ivgtt qd + CT	CT	14	③⑤
Li (2019a)	40/40	48/32	60.12 ± 5.34/61.58 ± 5.69	NR	Shenfu injection 40 ml ivgtt qd + CT	CT	90	①②③⑤
Mao (2016)	100/100	116/84	46–77	NR	Shenfu injection 40 ml ivgtt qd + CT	CT	5–10	①②④
Xiu and Chen (2015)	23/25	25/23	65.5 ± 10.1/63.4 ± 9.8	6,8,9/8,10,7	Shenfu injection 50 ml ivgtt qd + CT	CT	14	②③⑤
Wu and Duan (2009)	33/29	31/31	71.48 ± 5.78/73.59 ± 6.96	6,27,0/5,24,0	Shenfu injection 50 ml ivgtt qd + CT	CT	14	①②③⑤
Luo et al. (2015)	24/24	29/19	53.4 ± 11.7/50.9 ± 12.5	0,16,8/0,13,11	Shenfu injection 50 ml ivgtt qd + CT	CT	7	②③⑤
Li et al. (2016b)	60/60	72/48	61.32 ± 8.61/59.32 ± 8.35	18,31,11/20,30,10	Shenmai injection 100 ml ivgtt qd + CT	CT	14	⑤
Shen (2012)	50/50	47/53	62.87 ± 10.45	NR	Shenmai injection 50 ml ivgtt qd + CT	CT	90	①⑤
Yang and Li (2012)	30/30	35/25	65.5 ± 3.29/67 ± 2.56	NR	Shenmai injection 100 ml ivgtt qd 30–40 mg/ml + CT	CT	7	①②
Cao (2012)	60/60	85/35	42–80	66,44,10	Shenmai injection 100 ml ivgtt qd + CT	CT	14	①②
Zhao et al. (2011a)	53/53	55/51	32–75/32–75	17,30,6/16,32,5	Shenmai injection 60 ml ivgtt qd + CT	CT	15	④
Li (2019a)	26/26	31/21	76.42 ± 3.45/77.54 ± 4.4	NR	Shenmai injection 50 ml ivgtt qd + CT	CT	NR	③
Zhao et al. (2012)	35/35	53/17	66.8 ± 8.4/67.3 ± 9	NR	Shenmai injection 60 ml ivgtt + CT	CT	7	①③
Zhu et al. (2008)	38/38	41/35	63.7 ± 4.3/64.5 ± 4.8	NR	Shenqi Fuzheng injection 250 ml ivgtt + CT	CT	20	⑤
Wu (2014)	40/40	55/25	64.8 ± 5.2/65.3 ± 5	0,24,16/0,26,14	Shenqi Fuzheng injection 250 ml ivgtt qd + CT	CT	21	①②⑤
Lu (2005)	30/30	51/9	39–76/40–72	NR	Shenqi Fuzheng injection 250 ml ivgtt qd + CT	CT	21	①②
Zhan et al. (2017)	30/30	37/23	58.16 ± 2.26/58.39 ± 1.69	3,22,5/3,23,4	Dazhuhongjingtian injection 10 ml ivgtt qd + CT	CT	10	②⑥
Tian et al. (2017)	30/30	34/26	64.3 ± 6.8/67.2 ± 5.4	NR	Dazhuhongjingtian injection 10 ml ivgtt qd + CT	CT	10	②③⑤
Yang et al. (2014)	60/60	84/36	65.9 ± 16.4/66.3 ± 16.9	14,35,11/12,34,10	Danshenduofenyansuan injection 200 mg ivgtt qd + CT	CT	42	①③
Xu (2016)	60/52	66/46	62.2 ± 7.0/61.25 ± 5.4	30,30,0/30,22,0	Danshenduofenyansuan injection 200 mg ivgtt qd + CT	CT	42	③
Zhang (2020)	50/50	74/26	66.53 ± 5.56/65.18 ± 5.43	11,31,8/12,30,8	Danshenduofenyansuan injection 200 mg ivgtt qd + CT	CT	30	①③
Zhang (2015b)	23/22	25/20	77.39 ± 6.3/74.4 ± 4.8	10,10,3/9,8,5	Danhong injection 30 ml ivgtt qd + CT	CT	7	①③
Wang (2012)	43/43	42/44	54–81/48–80	0,28,15/0,26,17	Danhong injection 30 ml ivgtt qd + CT	CT	28	②⑤⑥
Xing et al. (2009)	55/53	72/36	54.9 ± 12.6/55.4 ± 11.8	NR	Dengzhanxixin injection 250 ml ivgtt qd + CT	CT	14	①②③
Teng (2016)	22/22	23/21	56.31 ± 3.44/55.67 ± 3.37	7,15,0/9,13,0	Gualoupi injection 8 ml ivgtt qd + CT	CT	7	①③
Ni et al. (2020)	40/40	44/36	68.9 ± 5.2/68.1 ± 4.9	NR	Gualoupi injection 8 ml ivgtt qd + CT	CT	7	⑤
Han (2018)	30/30	37/33	57.26 ± 6.34/57.21 ± 6.25	18,12,0/17,13,0	Shenfu injection 40 ml ivgtt qd + CT	CT	7	①④
Zhou et al. (2002)	56/47	72/31	61.3 ± 5.7/59.4 ± 6.3	15,32,9/13,30,4	Huangqi injection 60 ml ivgtt qd + CT	CT	21	①
Huang et al. (1999)	40/40	45/35	42–82/44/80	10,18,12/9,20,11	Shengmai injection 20 ml ivgtt qd + CT	CT	14	①②
Zhao et al. (2011b)	36/36	44/28	60.6 ± 10.4	18,38,16	Shengmai injection NR ivgtt qd + CT	CT	21	①②③
Wang et al. (2011)	70/70	78/62	64.7 ± 8.2/65.4 ± 7.8	NR	Shuxuening injection 20 ml ivgtt qd + CT	CT	14	③
Zhang (2015a)	150/150	153/147	71.8 ± 4.0/74.0 ± 4.0	0,69,81/0,70,80	Shuxuetong injection 250 ml ivgtt qd + CT	CT	10	①②③⑤
Ren (2021)	40/40	45/35	74.48 ± 2.22/72.21 ± 2.15	0,30,10/0,29,11	Xinmailong injection 5 mg/kg ivgtt bid 20–40 drops per minute + CT	CT	14	①②
Wei et al. (2020)	40/40	40/40	53.64 ± 7.56/54.25 ± 6.41	14,26,0/13,27,0	Xinmailong injection 4 ml ivgtt bid 30 drops per minute + CT	CT	7	⑤
Kuang (2004)	60/60	61/59	45–75/46–75	0,40,20/0,39,21	Xuesaitong injection 500 mg ivgtt qd + CT	CT	14	①②
Guo et al. (2012)	58/58	62/54	66–79/66–81	31,27,0/25,33,0	Shenmai injection 30 ml ivgtt qd + CT	CT	15	①②③⑥

E, experimental group; C, control group; M, male; F, female; CT, conventional treatment; NR, not report; ivgtt, intravenous glucose tolerance test; qd, one time a day; bid, two times a day; ① Total effective rate; ② Adverse events; ③ left ventricular ejection fraction (LVEF); ④ N-terminal pro-brain natriuretic peptide (NT-proBNP); ⑤brain natriuretic peptide (BNP); ⑥6-min walk distance (6MWT).

### Quality Evaluation

**FIGURE 2:**
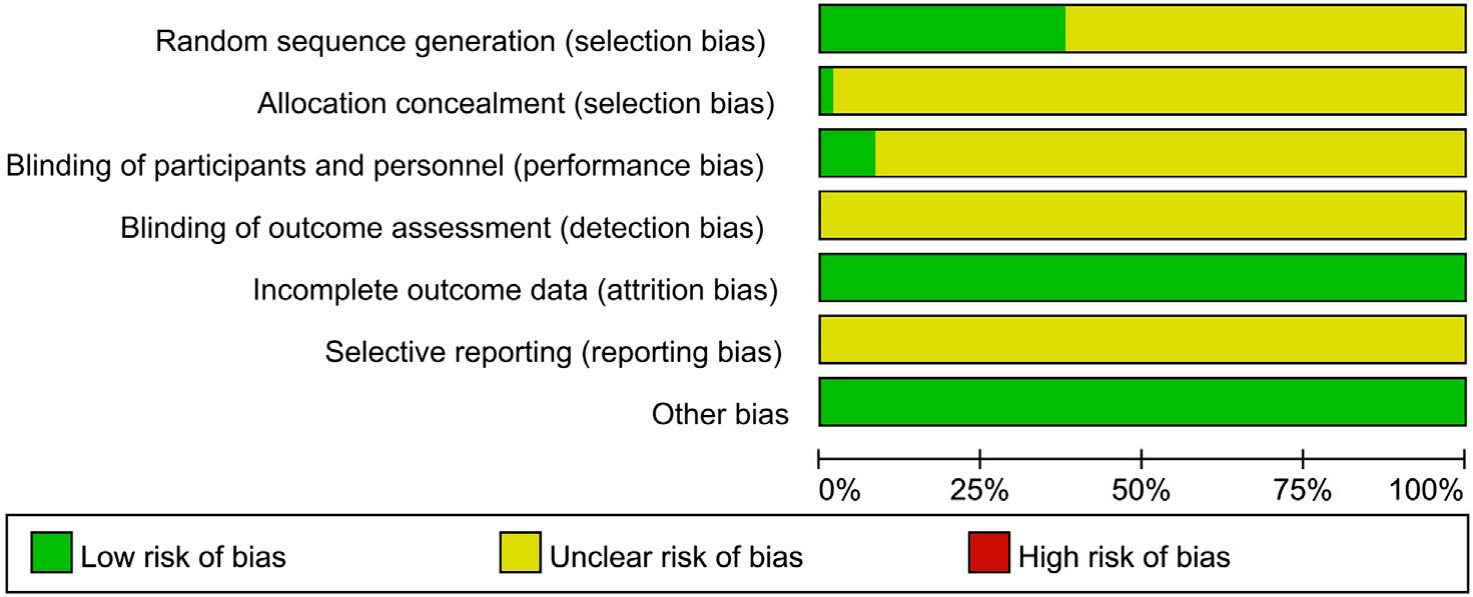
Risk of bias graph.

The detailed risk of bias assessments of the included studies is summarized in Figure 2 and Supplementary Figure S2. (1) Selective bias (random sequence generation and allocation concealment): The randomization of 12 RCTs (Hu et al., 2009; Zhu and Han, 2014; Xian et al., 2016; Xu and Cao, 2019; Shi et al., 2016; Wu et al., 2017; Wang et al., 2019; Shi et al., 2016; Wu et al., 2017; Wang et al., 2019; Shi et al., 2016; Wu et al., 2017; Wang et al., 2019; Shi et al., 2016; Wu et al., 2017; Wang et al., 2019; Shi et al., 2016; Wu et al., 2017; Wang et al., 2019; Shi et al., 2016; Wu et al., 2017; Wang et al., 2019; Shi et al., 2016; Wu et al., 2017; Wang et al., 2019; Shi et al., 2016; Wu et al., 2017; Wang et al., 2019; Shi et al., 2016; Wu et al., 2017; Wang et al., 2019) was generated *via* random number table, and three studies (Huang et al., 1999; Hu et al., 2009; Xian et al., 2016) *via* computer randomization, and two studies (Li et al., 2016a; Tian et al., 2017) *via* random parallel grouping method, and one study (Shen, 2012) *via* dynamic random grouping; therefore, the risk of selection bias was considered low. The remaining RCTs referred to only random grouping, and the risk of selection bias was considered unclear. (2) Performance bias (blinding of the participants and personnel): Three studies (Pan et al., 2005; Wang et al., 2011; Xian et al., 2016) were double-blind, and two studies (Dong, 2012; Luo et al., 2015) were single-blind, which were considered low risk. Other studies did not provide information on blinding, so the performance bias was evaluated as unclear risk. (3) Detection bias: There was not enough information to evaluate its risk level; therefore, the risk is unclear. (4) Attrition bias: None of the included RCTs had incomplete data, so the risk of attrition bias was considered “low.” (5) Reporting bias: Taking into account the inability to acquire a complete implementation scheme, the risk of reporting bias was considered “unclear.” (6) Other bias: The risk of this bias was considered “low,” because no other obvious bias was observed in all studies.

### Network Meta-Analysis in CHD Complicated With HF

**FIGURE 3:**
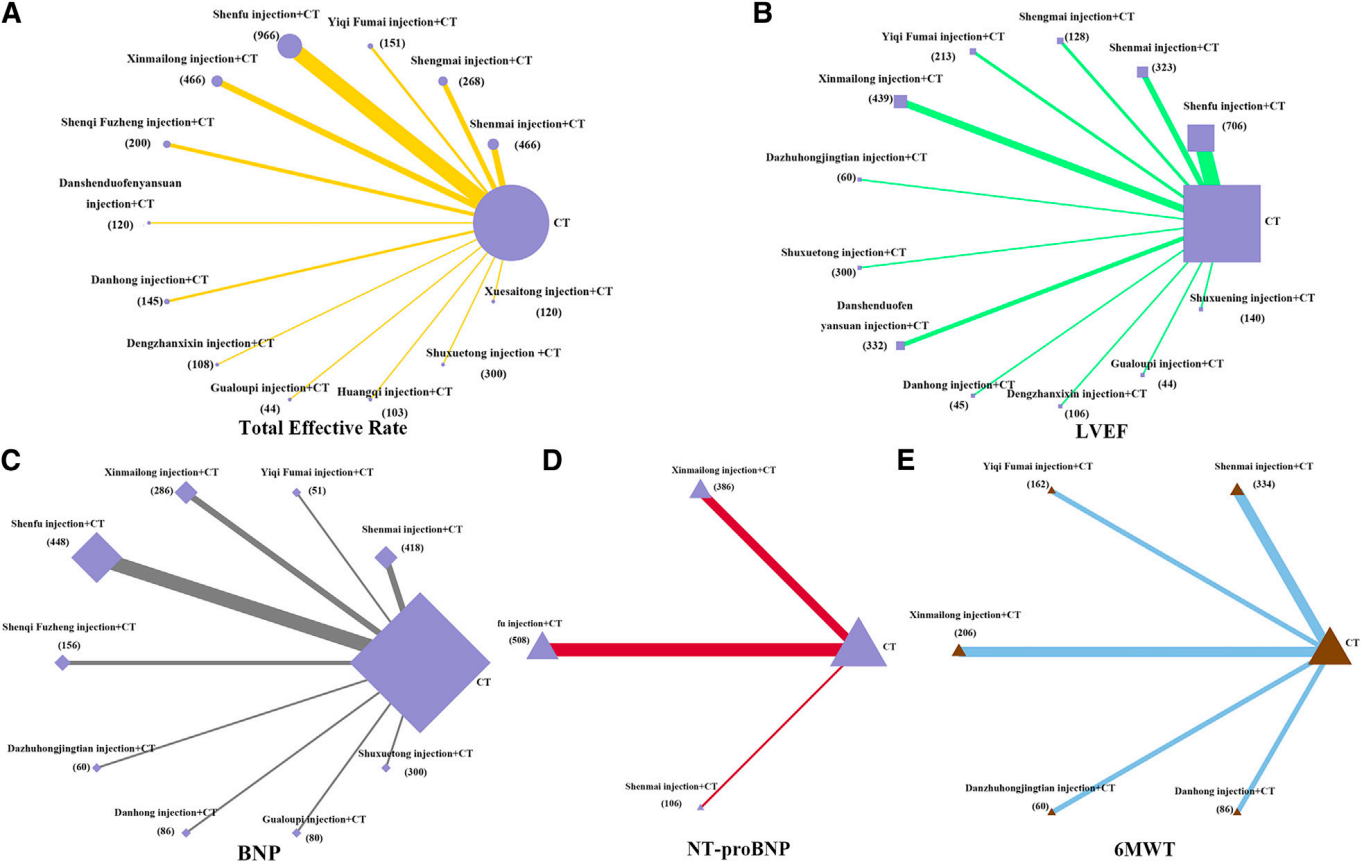
Network diagrams of comparisons on different outcomes of treatments in different groups of patients with CHD-HF. **(A)** total effective rate; **(B)** LVEF; **(C)** BNP; **(D)** NT-proBNP; **(E)** 6MWT. Each node represents a type of treatment. The node size is proportional to the total number of patients receiving a treatment (in brackets). Each line represents a type of head-to-head comparison. The width of lines is proportional to the number of trials comparing the connected treatments.

**FIGURE 4:**
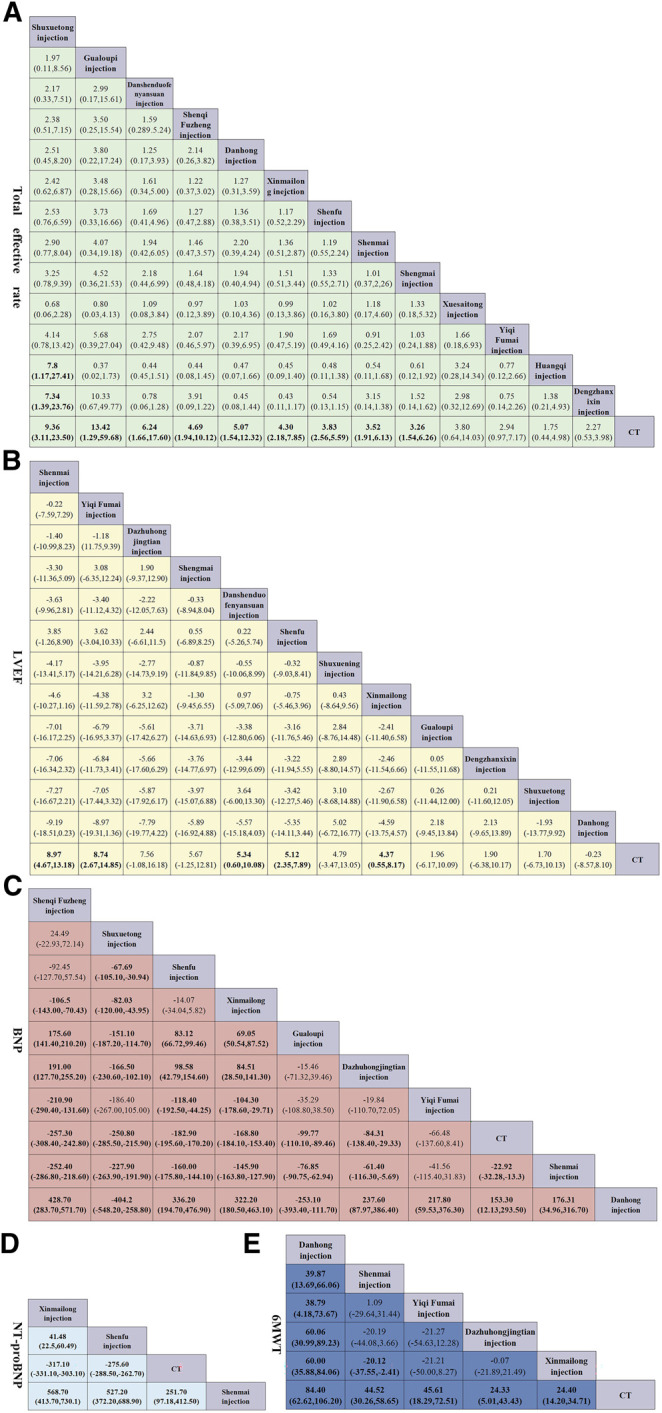
Pooled estimates of the network meta-analysis. **(A)** Pooled odd ratios (95% credible intervals) for the total effective rate. **(B)** Pooled odd ratios (95% credible intervals) for LVEF. **(C)** Pooled odd ratios (95% credible intervals) for NT-proBNP. **(D)** Pooled odd ratios (95% credible intervals) for BNP. **(E)** Pooled odd ratios (95% credible intervals) for 6MWT. Data in each cell are hazard or odds ratios (95% credible intervals) for the comparison of row-defining treatment versus column-defining treatment. Significant results are in bold. All the TCMIs based on CT.

Network meta-analysis included 13 treatments for the total effective rate, 12 treatments for LVEF, three treatments for NT-proBNP, nine treatments for BNP, and five treatments for 6MWT (Figure 3). In terms of the total effectiveness rate (Figure 4A), Shuxuetong injection was superior to all other therapies [vs. Huangqi injection (OR: 7.8, CI: 1.17–27.41), vs. Dengzhanxixin injection (OR: 7.34, CI: 1.39–23.76) and vs. CT (OR: 9.36, CI: 3.11–23.50)]. Compared to CT alone, CT combinations with the following TCMIs were significantly more effective: Gualoupi injection (OR: 13.42, CI: 1.29–59.68), Danshenduofenyansuan injection (OR: 6.24, CI: 1.66–17.60), Shenqi Fuzheng injection (OR: 4.69, CI: 1.94–10.12), Danhong injection (OR: 5.07, CI: 1.54–12.32), Xinmailong injection (OR: 4.30, CI: 2.18–7.85), Shenfu injection (OR: 3.83, CI: 2.56–5.59), Shenmai injection (OR: 3.52, CI: 1.91–6.13), and Shengmai injection (OR: 3.26, CI: 1.54–6.26).

For LVEF (Figure 4B), when compared to CT alone, Shenmai injection yielded best results (OR: 8.97, CI: 4.67–13.18); significant improvements were also found for Yiqi Fumai injection (OR: 8.74, CI: 2.67–4.85), Danshenduofenyansuan injection (OR: 5.34, CI: 0.60–10.08), Shenfu injection (OR: 5.12, CI: 2.35–7.89), and Xinmailong injection (OR: 4.37, CI: 0.55–8.17).

For NT-proBNP (Figure 4C), all the compared therapies significant differed from each other. Xinmailong injection, vs. Shenfu injection (OR: 41.48, CI: 22.50–60.49), and vs. CT (OR: −317.70, CI: −331.10–303.10). Shenfu injection with CT was superior to CT alone (OR: −275.60, CI: −288.50–262.70), whereas Shenmai injection with CT was not different from CT only.

For BNP (Figure 4D), Shenqi Fuzheng injection + CT was the best of all therapies [vs. Xinmailong injection (OR: 106.50, CI: −143.00–70.43), vs. Gualoupi injection (OR: 175.60, CI: 141.40–210.20); vs. Dazhuhongjingtian injection (OR: 191.00, CI: 127.70–255.20), vs. Yiqi Fumai injection (OR: −210.90, CI: −290.40–131.60), vs. CT (OR: −257.30,CI: −308.40–242.80), vs. Shenmai injection (OR: −252.40, CI: −286.80–218.60), vs. Danhong injection (OR: 428.70, CI: 283.70–517.70)]. The curative effect is in order for Xinmailong injection, Gualoupi injection, Dazhuhongjingtian injection, Yiqi Fumai injection, CT alone, Shenmai injection, and Danhong injection. Also, Shenmai injection and Danhong injection were not better than CT alone.

For 6MWT (Figure 4E), all TCMIs were superior to CT alone. Of those, Danhong injection was the best [vs. Shenmai injection (OR: 39.87, CI: 13.69–66.06); vs. Yiqi Fumai injection (OR: 38.79, CI: 4.18–73.67); vs. Dazhuhongjingtian injection (OR: 60.06, CI: 30.99–89.23); vs. Xinmailong (OR: 60.00, CI: 35.88–84.06); vs. CT (OR: 84.40, CI: 62.62–106.20)].

### Rank Probabilities

**FIGURE 5:**
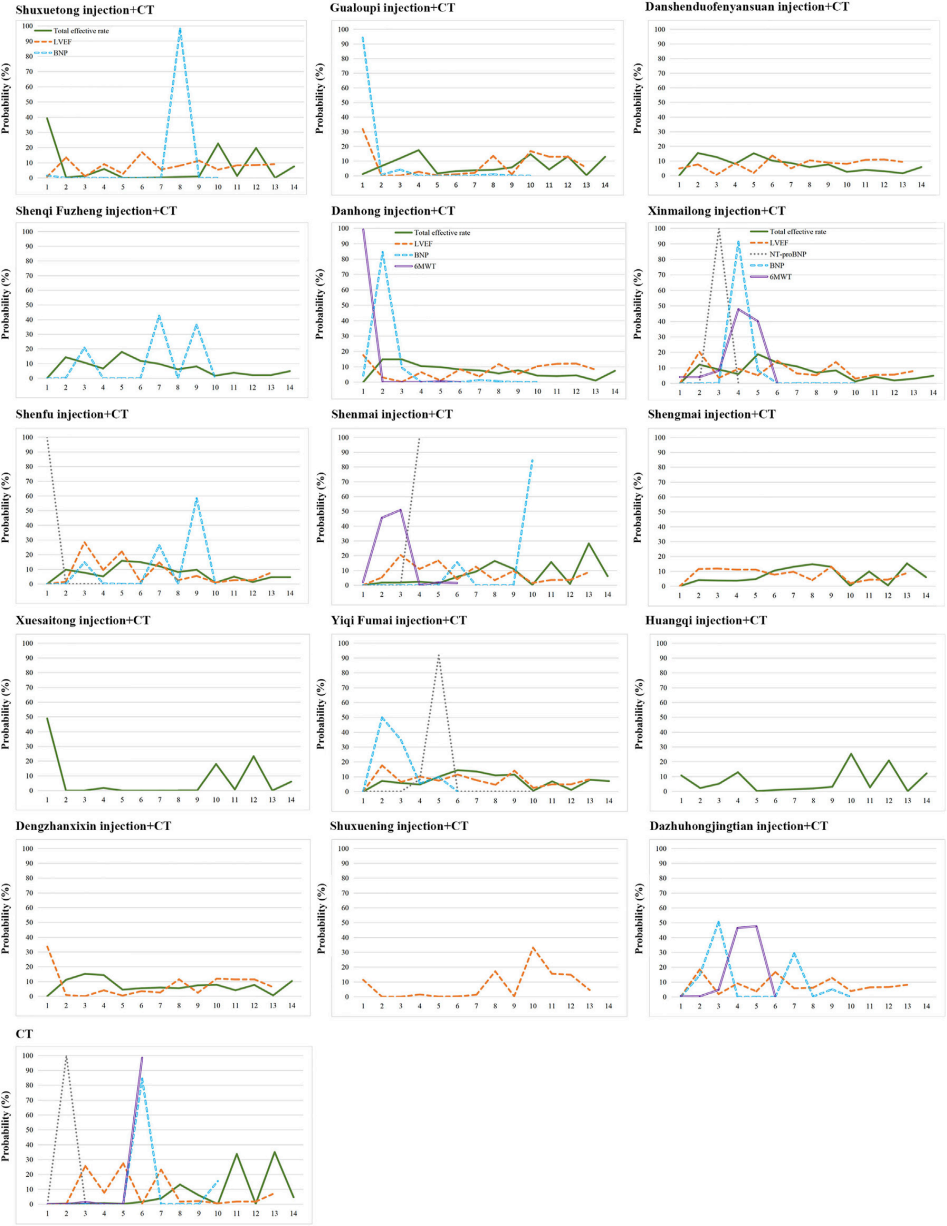
Bayesian ranking profiles of comparable treatments on efficacy for patients with CHD-HF.

Figure 5 shows the Bayesian ranking profiles of the compared treatments with the detail ranking results summarized in Supplementary Tables S5–S9. Shuxuetong injection was most likely to be ranked first for the total effectiveness rate (cumulative probability 39.24%), Shenmai injection for LVEF (20.37%), Xinmailong injection for NT-proBNP (99.99%), Shenqi Fuzheng injection for BNP (42.55%), and Danhong injection for 6MWT (99.24%).

### Assessment of Heterogeneity and Inconsistency

Forest plots for the four available pairwise comparisons that include heterogeneity estimates are shown in Supplementary Figure S3. Our assessment suggested minimal heterogeneity (I^2^ = 0.00%) in half of all comparisons regarding different outcomes. However, moderate to high heterogeneity was detected in the following comparisons:

Shenfu injection vs. CT alone for LVEF (93.24%) and BNP (98.29%); Shenmai injection vs. CT alone for LVEF (98.89%), NT-proBNP (99.53%), and BNP (98.51%); Shengmai injection vs. CT alone for LVEF (97.04%) and 6MWT (39.21%); Yiqi Fumai injection vs. CT alone for LVEF (78.35%); Xinmailong injection vs. CT alone for LVEF (99.58%), NT-proBNP (99.24%), and 6MWT (58.09%); and Shenqi Fuzheng injection vs. CT alone for BNP (98.44%). The fit of the consistency model was similar or better than that of the inconsistency model (Supplementary Table S10).

### Adverse Events

Thirty-one reports (Shi et al., 2016; Wu et al., 2017; Wang et al., 2019; Shi et al., 2016; Wu et al., 2017; Wang et al., 2019; Shi et al., 2016; Wu et al., 2017; Wang et al., 2019; Shi et al., 2016; Wu et al., 2017; Wang et al., 2019; Shi et al., 2016; Wu et al., 2017; Wang et al., 2019; Shi et al., 2016; Wu et al., 2017; Wang et al., 2019; Gong et al., 2018; Ji, 2019; He, 2016; Wang et al., 2016; Zhou et al., 2013; Wu, 2016; Dong, 2012; Li et al., 2018; Wang and Jang, 2018; Li, 2019a; Zhou and Luo, 2020; Wu and Duan, 2009; Luo et al., 2015; Zhu et al., 2008; Zhao et al., 2011a; Zhao et al., 2012; Li, 2019b; Yang et al., 2014; Zhang, 2020; Teng, 2016; Ni et al., 2020; Huang et al., 1999; Zhou et al., 2002; Wang et al., 2011; Zhang, 2015b) considered the occurrence of ADRs; of those, 25 reports listed no ADRs, and 6 records described specific ADRs (Huang et al., 1999; Xin and Shan, 2012; Zhao et al., 2012; Shi et al., 2016; Wang and Jang, 2018; Ni et al., 2020), such as renal dysfunction, liver dysfunction, urinary system infection or urine protein, etc. (Table 2). The ADR rates of Shenfu injection, Shengmai injection, Xinmailong injection, Dazhuhongjingtian injection, and CT alone were 21.05, 5.56, 5.26, 6.67, and 7.28%, respectively.

**TABLE 2:** Occurrence of adverse reactions of TCMIs.

No. of studies	2	1	2	1	6
Sample size	114	36	95	30	261
Treatments	Shenfu injection	Shengmai injection	Xinmailong injection	Dazhuhongjingtian injection	CT
Renal dysfunction	**6**	0	0	0	**4**
Liver dysfunction	**1**	0	0	0	**2**
Urinay system infection	**5**	0	0	0	**3**
Urine protein	**1**	0	0	0	**1**
Pulmonary infection	**1**	0	0	0	0
Anemia	**1**	0	0	0	0
Hypoglycemia	**1**	0	0	0	0
Chills	**2**	0	0	0	0
Erythra	**1**	0	0	0	0
Diarrhea	0	0	0	0	**1**
Ureteral calculi cut into stone	**1**	0	0	0	0
Nausea	**2**	**1**	**1**	0	**2**
Mouth dryness	0	0	**1**	0	**1**
Flush face	0	0	**1**	0	0
Dizziness	**1**	0	**1**	0	0
Allergy	**1**	0	0	0	0
Hemorrhage	0	0	0	**2**	**3**
Abdominal distension	0	**1**	0	0	0
Headache	0	0	**1**	0	**2**

Significant results are in bold.

### Sensitivity Analysis

A total of 2,959 patients in 22 trials with case numbers ≥100 trials (Zhu and Han, 2014; Xian et al., 2016; Xu and Cao, 2019; Zhu and Han, 2014; Xian et al., 2016; Xu and Cao, 2019; Zhu and Han, 2014; Xian et al., 2016; Xu and Cao, 2019; Zhu and Han, 2014; Xian et al., 2016; Xu and Cao, 2019; Zhu and Han, 2014; Xian et al., 2016; Xu and Cao, 2019; Zhu and Han, 2014; Xian et al., 2016; Xu and Cao, 2019; Zhu and Han, 2014; Xian et al., 2016; Xu and Cao, 2019; Zhu and Han, 2014; Xian et al., 2016; Xu and Cao, 2019; Zhu and Han, 2014; Xian et al., 2016; Xu and Cao, 2019; Zhu and Han, 2014; Xian et al., 2016; Xu and Cao, 2019; Zhu and Han, 2014; Xian et al., 2016; Xu and Cao, 2019; Yang et al., 2014; Xu, 2016; Zhang, 2020; Yang et al., 2014; Xu, 2016; Zhang, 2020; Yang et al., 2014; Xu, 2016; Zhang, 2020; Yang et al., 2014; Xu, 2016; Zhang, 2020; Yang et al., 2014; Xu, 2016; Zhang, 2020; Yang et al., 2014; Xu, 2016; Zhang, 2020) were included in sensitivity analysis. The results did not show any obvious deviations from the original network meta-analysis (Supplementary Figures S4–S5). Among the findings, Yiqi Fumai injection yielded the best therapeutic effects for LVEF and 6MWT, which were only slightly different from the original meta-analysis.

## Discussion

In this systematic review and network meta-analysis, we comprehensively summarize the efficacy and safety of different TCMI treatments in patients with CHD-HF. The results suggest that (1) many TCMI combined with CT are superior to CT alone in the total effectiveness rate, LVEF, NT-proBNP, BNP, and 6MWT, although CT alone was superior to some TCMIs combined with CT in improving NT-BNP and BNP indices; (2) Shuxuetong injection, Shenmai injection, Xinmailong injection, Shenqi Fuzheng injection, and Danhong injection had the best curative effect when measured by the total effectiveness rate, LVEF, NT-proBNP, BNP, and 6MWT, respectively; (3) Shuxuetong injection, Gualoupi injection, and Danshenduofenyansuan injection (which stimulate blood circulation and prevent blood stasis) were consistent in improving the total effectiveness rate, but not LVEF; (4) Shenmai injection, Yiqi Fumai injection, and Shengmai injection (which invigorate qi) were consistent in ameliorating LVEF; (5) Shenfu injection (which revives yang) and Xinmailong injection (qi-invigorating and blood-activating) were consistent in improving NT-proBNP and BNP; (6) Danhong injection (which invigorates blood circulation) and Shenmai injection (which supplements qi and nourishes yin) were beneficial for 6MWT.

The safety of TCMIs has always been of concern. A total of 31 studies in our network meta-analysis considered safety issues, and most did not report any serious ADRs. The common side effects were nausea, mouth dryness, and dizziness. Renal dysfunction, liver dysfunction, urinary system infection, urine protein, pulmonary infection, anemia, hypoglycemia, and chills were reported occasionally. These discomforts could be effectively relieved by symptomatic treatments. Nevertheless, clinicians should keep in mind the possibility of ADRs when prescribing TCMI treatments. In our comparisons, Shenfu injection had the least favorable safety profile.

Conditions of patients with CHD-HF are often serious. Despite advances in treatments, the 5-years and 10-years survival rates are still estimated to be 50% and 10%, respectively, and the readmission rates continue to rise (Ren, 2021). In China, TCMIs are approved by the China Food and Drug Administration and are widely used in patients hospitalized due to CHD-HF. Some studies have shown that TCMIs combined with CT had some advantages. For example, Shenfu injections were reported to improve the NYHA functional classification, TCM syndrome score, 6MWT and SF-36 health survey score, increase the number of CD + 34 stem cells in the peripheral blood, and promote mobilization of bone marrow stem cells (Hu et al., 2009; Xian et al., 2016; Wang et al., 2019). Wu et al. (2017) indicated that Xinmailong injection can effectively inhibit inflammatory reactions and improve the indices of cardiac function in patients with CHD-HF. Basic studies revealed that Shenfu injection opposes heart failure through anti-apoptosis, anti-oxidation, and reduction of myocardial fibrosis (Ni et al., 2017; Yan et al., 2018; Wu et al., 2019). Xinmailong injection could notably reduce the production of reactive oxygen species and enhance the protein expressions of antioxidant enzymes, thereby exerting therapeutic effects on the cardiovascular system (Li et al., 2017). Shengmai injection may attenuate oxidative stress-induced damage in cardiomyocytes potentially through the AKT and ERK1/2 pathways that protect against heart failure (Zhu et al., 2019).

Our sensitivity analysis showed that the overall results remained relatively robust when the trials were restricted to case numbers ≥100. The SUCRA rankings for LVEF and BNP of Shenfu injection and Shenmai injection had differences, which could be due to the low number of studies that considered these outcomes.

### Limitations

Our study had several limitations. First, our analysis could be complicated by various confounding factors beyond our control, because most treatments were indirectly compared and most direct evidence was derived from one trial in the present network. Second, despite our best efforts, the included RCTs were of relatively poor quality. For example, although all trials reported that patients were randomly assigned into different groups, only some of 61 RCTs described the specific methods of generating random sequences, such as a random number table or a random parallel grouping. Only five studies mentioned the blinding method, and most trials were low sample size tests with positive findings which are particularly prone to various biases. Third, most of the included studies have not been registered.

### Implications

By synthesizing all evidence from multiple RCTs, this review helps to identify current problems and areas worthy of improvement. Due to the poor quality of the included studies, the evidence obtained from our network meta-analysis is not sufficient for a comprehensive comparison of different TCMI combinations with various CTs for treatment of CHD-HF. Based on our findings, we propose the following two recommendations for further studies on TCMI-CT in the treatment of CHD-HF: (1) clinical trials should be prospectively registered in recognized clinical trials registry platforms; and (2) the quality of study designs should be improved, including randomization, allocation concealment, and blinding.

## Conclusion

In this network meta-analysis, the TCMIs known for promoting blood circulation and preventing blood stasis, such as shuxuetong injection, danshenduofenyansuan injection, improved cardiac function, and clinical symptoms when compared in CTs; the qi-invigorating, such as Shenqi fuzheng injection, Shenmai injection, and Xinmailong injection improve the indices of LVEF, NT-proBNP, BNP, and 6MWT. Our analysis also revealed that Shenfu injection has obvious side effects, which should be paid more attention to in clinical applications. Whereas the high risk of bias and low quality of the available trials may limit the reliability of our trial comparisons, our analysis clearly reveals the need for more well-designed clinical studies with improved sample sizes and quality of data processing.

More clinical studies with well-designed, reasonable samples and good method quality are needed in the future.

## Data Availability

The original contributions presented in the study are included in the article/Supplementary Material, and further inquiries can be directed to the corresponding author/s.

## Abbreviation

BNP, brain natriuretic peptide; CHD, coronary heart disease; HF, heart failure; LVEF, left ventricular ejection fraction; NT-proBNP, N-terminal pro-brain natriuretic peptide; NYHA, New York Heart Association functional class; TCMIs, traditional Chinese medicine injections; RCTs, randomised controlled trials; 6MWT, 6-min walk test.
