# Supplementary material for: Efficacy and Safety of TCMI in Patients With Combined Coronary Heart Disease and Heart Failure: A Systematic Review and Network Meta-Analysis
Source: Front Pharmacol. 2021 Nov 23;12:741261. doi: 10.3389/fphar.2021.741261 (PMC8652334; doi:10.3389/fphar.2021.741261)
Supplement: Supplementary file 1 [file DataSheet1.docx]

***Supplementary materials***

***Supplementary table S1***

The PRISMA checklist of this meta-analysis

| **Section and Topic** | **Item #** | **Checklist item** | **Location where item is reported** |
| --- | --- | --- | --- |
| **TITLE** | | |  |
| Title | 1 | Identify the report as a systematic review. | 1 |
| **ABSTRACT** | | |  |
| Abstract | 2 | See the PRISMA 2020 for Abstracts checklist. | 1 |
| **INTRODUCTION** | | |  |
| Rationale | 3 | Describe the rationale for the review in the context of existing knowledge. | 2 |
| Objectives | 4 | Provide an explicit statement of the objective(s) or question(s) the review addresses. | 3 |
| **METHODS** | | |  |
| Eligibility criteria | 5 | Specify the inclusion and exclusion criteria for the review and how studies were grouped for the syntheses. | 4 |
| Information sources | 6 | Specify all databases, registers, websites, organisations, reference lists and other sources searched or consulted to identify studies. Specify the date when each source was last searched or consulted. | 3-4 |
| Search strategy | 7 | Present the full search strategies for all databases, registers and websites, including any filters and limits used. | Supplementary table S2 |
| Selection process | 8 | Specify the methods used to decide whether a study met the inclusion criteria of the review, including how many reviewers screened each record and each report retrieved, whether they worked independently, and if applicable, details of automation tools used in the process. | 4 |
| Data collection process | 9 | Specify the methods used to collect data from reports, including how many reviewers collected data from each report, whether they worked independently, any processes for obtaining or confirming data from study investigators, and if applicable, details of automation tools used in the process. | 5 |
| Data items | 10a | List and define all outcomes for which data were sought. Specify whether all results that were compatible with each outcome domain in each study were sought (e.g. for all measures, time points, analyses), and if not, the methods used to decide which results to collect. | 5 |
|  | 10b | List and define all other variables for which data were sought (e.g. participant and intervention characteristics, funding sources). Describe any assumptions made about any missing or unclear information. | 5 |
| Study risk of bias assessment | 11 | Specify the methods used to assess risk of bias in the included studies, including details of the tool(s) used, how many reviewers assessed each study and whether they worked independently, and if applicable, details of automation tools used in the process. | 5-6 |
| Effect measures | 12 | Specify for each outcome the effect measure(s) (e.g. risk ratio, mean difference) used in the synthesis or presentation of results. | 6 |
| Synthesis methods | 13a | Describe the processes used to decide which studies were eligible for each synthesis (e.g. tabulating the study intervention characteristics and comparing against the planned groups for each synthesis (item #5)). | 6 |
|  | 13b | Describe any methods required to prepare the data for presentation or synthesis, such as handling of missing summary statistics, or data conversions. | 6 |
|  | 13c | Describe any methods used to tabulate or visually display results of individual studies and syntheses. | 6 |
|  | 13d | Describe any methods used to synthesize results and provide a rationale for the choice(s). If meta-analysis was performed, describe the model(s), method(s) to identify the presence and extent of statistical heterogeneity, and software package(s) used. | 6 |
|  | 13e | Describe any methods used to explore possible causes of heterogeneity among study results (e.g. subgroup analysis, meta-regression). | 6 |
|  | 13f | Describe any sensitivity analyses conducted to assess robustness of the synthesized results. | 6 |
| Reporting bias assessment | 14 | Describe any methods used to assess risk of bias due to missing results in a synthesis (arising from reporting biases). | 6 |
| Certainty assessment | 15 | Describe any methods used to assess certainty (or confidence) in the body of evidence for an outcome. | 6 |
| **RESULTS** | | |  |
| Study selection | 16a | Describe the results of the search and selection process, from the number of records identified in the search to the number of studies included in the review, ideally using a flow diagram. | 7 |
|  | 16b | Cite studies that might appear to meet the inclusion criteria, but which were excluded, and explain why they were excluded. | Fig1 |
| Study characteristics | 17 | Cite each included study and present its characteristics. | 8 |
| Risk of bias in studies | 18 | Present assessments of risk of bias for each included study. | 7-8 |
| Results of individual studies | 19 | For all outcomes, present, for each study: (a) summary statistics for each group (where appropriate) and (b) an effect estimate and its precision (e.g. confidence/credible interval), ideally using structured tables or plots. | Supplementary figure S3 |
| Results of syntheses | 20a | For each synthesis, briefly summarise the characteristics and risk of bias among contributing studies. | 7 |
|  | 20b | Present results of all statistical syntheses conducted. If meta-analysis was done, present for each the summary estimate and its precision (e.g. confidence/credible interval) and measures of statistical heterogeneity. If comparing groups, describe the direction of the effect. | 9 |
|  | 20c | Present results of all investigations of possible causes of heterogeneity among study results. | 9 |
|  | 20d | Present results of all sensitivity analyses conducted to assess the robustness of the synthesized results. | 9Supplementary S4&S5 |
| Reporting biases | 21 | Present assessments of risk of bias due to missing results (arising from reporting biases) for each synthesis assessed. | 9 |
| Certainty of evidence | 22 | Present assessments of certainty (or confidence) in the body of evidence for each outcome assessed. | Supplementary S3 |
| **DISCUSSION** | | |  |
| Discussion | 23a | Provide a general interpretation of the results in the context of other evidence. | 10 |
|  | 23b | Discuss any limitations of the evidence included in the review. | 11 |
|  | 23c | Discuss any limitations of the review processes used. | 11-12 |
|  | 23d | Discuss implications of the results for practice, policy, and future research. | 12 |
| **OTHER INFORMATION** | | |  |
| Registration and protocol | 24a | Provide registration information for the review, including register name and registration number, or state that the review was not registered. | 3 |
|  | 24b | Indicate where the review protocol can be accessed, or state that a protocol was not prepared. | 3 |
|  | 24c | Describe and explain any amendments to information provided at registration or in the protocol. | 3 |
| Support | 25 | Describe sources of financial or non-financial support for the review, and the role of the funders or sponsors in the review. | 17 |
| Competing interests | 26 | Declare any competing interests of review authors. | 17 |
| Availability of data, code and other materials | 27 | Report which of the following are publicly available and where they can be found: template data collection forms; data extracted from included studies; data used for all analyses; analytic code; any other materials used in the review. | 17 |

***Supplementary table S2*** The details of search terms and literature search strategy

Take searching PubMed as an example, the search terms and strategies are as follows:

| #1 Coronary Diseases [MeSH Terms]  #2 Coronary Diseases [Title/Abstract]  #3 Coronary Heart Disease [MeSH Terms]  #4 Coronary Heart Disease [Title/Abstract]  #5 #1 OR #2 OR #3 OR #4  #6 Heart failure [MeSH Terms]  #7 Heart failure [Title/Abstract]  #8 #6 OR #7  #9 Traditional Chinese medicine [MeSH Terms]  #10 Traditional Chinese medicine [Title/Abstract]  #11 Injection [MeSH Terms]  #12 Injection [Title/Abstract]  #13 Injectable [MeSH Terms]  #14 Injectable [Title/Abstract]  #15 #8 OR #9 OR #10 OR #11 OR #12 OR #13 OR #14  #16 Randomized Controlled Trial [Publication Type]  #17 Controlled Clinical Trial [Publication Type]  #18 random* [All Fields]  #19 #16 OR #17 OR #18  #20 #5AND #8 AND #15 AND #19 |
| --- |

***Supplementary*** ***table S3*** The details of TCMIs of all the included studies

| **Study** | **Formulation** | **Source** | **Species** | **Quality control reported? (Y/N)** | **Chemial analysis reported? (Y/N)** |
| --- | --- | --- | --- | --- | --- |
| Hu et al. (2009) | Shenfu injection | Sichuan Yaan SanJiu Pharmaceutical Co., Ltd | Root of *Panax ginseng C. A. Mey.,*Subroot of *Aconitum Carmichaelii Debx* | Y-Prepared according to Chinese pharmacopeia | Y-Spectrophotometry |
| Xian et al. (2016) | Shenmai injection | Qingchunbao Pharmaceutical Co., Ltd (Hangzhou, China) | Root of *Panax ginseng C. A. Mey.,* Root of *Ophiopogon japonicus （L.f） Ker-GawL* | Y-Prepared according to Chinese pharmacopeia | Y-HPLC |
| Xin et al. (2012) | Shengmai injection | Jilin Jian Yisheng Pharmaceutical Co.,Ltd. | Root of *Panax ginseng C.A.Mey./Codonopsis pilosula (Franch.)Nannf.,*Root of *Ophiopogon japonicus （L.f） Ker-Gaw,*Fruit of *Schisandra chinensis（Turcz.）Baill.* | Y-Prepared according to Chinese pharmacopeia | Y-HPLC |
| Zhu et al. (2014) | Qiqi Fumai injection | Not mentioned | Root of *Panax ginseng C.A.Mey./Codonopsis pilosula (Franch.)Nannf.,*Root of *Ophiopogon japonicus （L.f） Ker-Gaw,*Fruit of *Schisandra chinensis（Turcz.）Baill.* | － | － |
| Feng (2013) | Qiqi Fumai injection | Not mentioned | Root of *Panax ginseng C.A.Mey./Codonopsis pilosula (Franch.)Nannf.,*Root of *Ophiopogon japonicus （L.f） Ker-Gaw,*Fruit of *Schisandra chinensis（Turcz.）Baill.* | － | － |
| Yuan et al. (2012) | Qiqi Fumai injection | Not mentioned | Root of *Panax ginseng C.A.Mey./Codonopsis pilosula (Franch.)Nannf.,*Root of *Ophiopogon japonicus （L.f） Ker-Gaw,*Fruit of *Schisandra chinensis（Turcz.）Baill.* | － | － |
| Pan et al. (2005) | Shengmai injection | Shanghai Hehuang Pharmaceutical Co., Ltd | Root of *Panax ginseng C.A.Mey./Codonopsis pilosula (Franch.)Nannf.,*Root of *Ophiopogon japonicus （L.f） Ker-Gaw,*Fruit of *Schisandra chinensis（Turcz.）Baill.* | Y-Prepared according to Chinese pharmacopeia | Y-HPLC |
| Wang et al. (2019) | Shenfu injection | Not mentioned | Root of *Panax ginseng C. A. Mey.,*Subroot of *Aconitum Carmichaelii Debx* | － | － |
| Xu et al. (2019) | Xinmailong injection | Yunnan Tengyao Pharmaceutical Co., Ltd | Extractum Xinmailong | Y-Prepared according to Chinese pharmacopeia(2005) | Y-HPLC |
| Wu et al. (2017) | Xinmailong injection | Yunnan Tengyao Pharmaceutical Co., Ltd | Extractum Xinmailong | Y-Prepared according to Chinese pharmacopeia(2005) | Y-HPLC |
| Shi et al. (2016) | Xinmailong injection | Yunnan Tengyao Pharmaceutical Co., Ltd | Extractum Xinmailong | Y-Prepared according to Chinese pharmacopeia(2005) | Y-HPLC |
| Shen et al. (2017) | Xinmailong injection | Yunnan Tengyao Pharmaceutical Co., Ltd | Extractum Xinmailong | Y-Prepared according to Chinese pharmacopeia(2005) | Y-HPLC |
| Li et al. (2016) | Xinmailong injection | Yunnan Tengyao Pharmaceutical Co., Ltd | Extractum Xinmailong | Y-Prepared according to Chinese pharmacopeia(2005) | Y-HPLC |
| Ji (2019) | Xinmailong injection | Yunnan Tengyao Pharmaceutical Co., Ltd | Extractum Xinmailong | Y-Prepared according to Chinese pharmacopeia(2005) | Y-HPLC |
| Gong et al. (2018) | Xinmailong injection | Yunnan Tengyao Pharmaceutical Co., Ltd | Extractum Xinmailong | Y-Prepared according to Chinese pharmacopeia(2005) | Y-HPLC |
| Wu et al. (2012) | Shenfu injection | Not mentioned | Root of *Panax ginseng C. A. Mey.,*Subroot of *Aconitum Carmichaelii Debx* | － | － |
| He et al. (2016) | Shenfu injection | Ya 'an Sanjiu Pharmaceutical Co., Ltd | Root of *Panax ginseng C. A. Mey.,*Subroot of *Aconitum Carmichaelii Debx* | Y-Prepared according to Chinese pharmacopeia | Y-Spectrophotometry |
| Wang et al. (2016) | Shenfu injection | Not mentioned | Root of *Panax ginseng C. A. Mey.,*Subroot of *Aconitum Carmichaelii Debx* | － | － |
| Yang (2009) | Shenfu injection | Ya 'an Sanjiu Pharmaceutical Co., Ltd | Root of *Panax ginseng C. A. Mey.,*Subroot of *Aconitum Carmichaelii Debx* | Y-Prepared according to Chinese pharmacopeia | Y-Spectrophotometry |
| Wu (2016) | Shenfu injection | Sanjiu pharmaceutical Co.,Ltd | Root of *Panax ginseng C. A. Mey.,*Subroot of *Aconitum Carmichaelii Debx* | Y-Prepared according to Chinese pharmacopeia | Y-Spectrophotometry |
| Zhou et al. (2013) | Shenfu injection | Not mentioned | Root of *Panax ginseng C. A. Mey.,*Subroot of *Aconitum Carmichaelii Debx* | － | － |
| Zhou et al. (2005) | Shenfu injection | Ya 'an Sanjiu Pharmaceutical Co., Ltd | Root of *Panax ginseng C. A. Mey.,*Subroot of *Aconitum Carmichaelii Debx* | Y-Prepared according to Chinese pharmacopeia | Y-Spectrophotometry |
| Wang et al. (2018) | Shenfu injection | Huarun Sanjiu (Ya 'an) Pharmaceutical Co., Ltd | Root of *Panax ginseng C. A. Mey.,*Subroot of *Aconitum Carmichaelii Debx* | Y-Prepared according to Chinese pharmacopeia | Y-Spectrophotometry |
| Zhou et al. (2020) | Shenfu injection | Ya 'an Sanjiu Pharmaceutical Co., Ltd | Root of *Panax ginseng C. A. Mey.,*Subroot of *Aconitum Carmichaelii Debx* | Y-Prepared according to Chinese pharmacopeia | Y-Spectrophotometry |
| Dong et al. (2012) | Shenfu injection | Ya 'an Sanjiu Pharmaceutical Co., Ltd | Root of *Panax ginseng C. A. Mey.,*Subroot of *Aconitum Carmichaelii Debx* | Y-Prepared according to Chinese pharmacopeia | Y-Spectrophotometry |
| Li et al. (2018) | Shenfu injection | Huarun Sanjiu Pharmaceutical Co., Ltd | Root of *Panax ginseng C. A. Mey.,*Subroot of *Aconitum Carmichaelii Debx* | Y-Prepared according to Chinese pharmacopeia | Y-Spectrophotometry |
| Li (2019) | Shenfu injection | Ya 'an Sanjiu Pharmaceutical Co., Ltd | Root of *Panax ginseng C. A. Mey.,*Subroot of *Aconitum Carmichaelii Debx* | Y-Prepared according to Chinese pharmacopeia | Y-Spectrophotometry |
| Mao (2016) | Shenfu injection | Not mentioned | Root of *Panax ginseng C. A. Mey.,*Subroot of *Aconitum Carmichaelii Debx* | － | － |
| Xiu et al. (2015) | Shenfu injection | Not mentioned | Root of *Panax ginseng C. A. Mey.,*Subroot of *Aconitum Carmichaelii Debx* | － | － |
| Duan (2009) | Shenfu injection | Shenzhen Southern Pharmaceutical Factory Ya 'an Sanjiu Pharmaceutical Co. Ltd | Root of *Panax ginseng C. A. Mey.,*Subroot of *Aconitum Carmichaelii Debx* | Y-Prepared according to Chinese pharmacopeia | Y-Spectrophotometry |
| Luo et al. (2015) | Shenfu injection | Not mentioned | Root of *Panax ginseng C. A. Mey.,*Subroot of *Aconitum Carmichaelii Debx* | － | － |
| Li et al. (2016) | Shenmai injection | Not mentioned | Root of *Panax ginseng C. A. Mey.,* Root of *Ophiopogon japonicus （L.f） Ker-GawL* | － | － |
| Shen (2012) | Shenmai injection | Not mentioned | Root of *Panax ginseng C. A. Mey.,* Root of *Ophiopogon japonicus （L.f） Ker-GawL* | － | － |
| Yang et al. (2012) | Shenmai injection | Zhengda Qingchunbao Pharmaceutical Co., Ltd | Root of *Panax ginseng C. A. Mey.,* Root of *Ophiopogon japonicus （L.f） Ker-GawL* | Y-Prepared according to Chinese pharmacopeia | Y-HPLC |
| Cao (2012) | Shenmai injection | Hebei Shenwei Pharmaceutical Co. Ltd | Root of *Panax ginseng C. A. Mey.,* Root of *Ophiopogon japonicus （L.f） Ker-GawL* | Y-Prepared according to Chinese pharmacopeia | Y-HPLC |
| Zhao et al. (2011) | Shenmai injection | Zhengda Qingchunbao Pharmaceutical Co., Ltd | Root of *Panax ginseng C. A. Mey.,* Root of *Ophiopogon japonicus （L.f） Ker-GawL* | Y-Prepared according to Chinese pharmacopeia | Y-HPLC |
| Li (2019) | Shenmai injection | Not mentioned | Root of *Panax ginseng C. A. Mey.,* Root of *Ophiopogon japonicus （L.f） Ker-GawL* | － | － |
| Zhao et al. (2012) | Shenmai injection | Not mentioned | Root of *Panax ginseng C. A. Mey.,* Root of *Ophiopogon japonicus （L.f） Ker-GawL* | － | － |
| Zhu et al. (2008) | Shenqi Fuzheng injection | Lizhu Group Limin Pharmaceutical Factory | Root of *Codonopsis pilosula (Franch.)Nannf.,*Root of *Astragalus membranaceus(Fisch.) Bge.var.mongholicus(Bge.)* | Y-Prepared according to Chinese pharmacopeia(2000) | Y-Spectrophotometry |
| Wu (2014) | Shenqi Fuzheng injection | Shenzhen Lizhu Group pharmaceutical Co., Ltd | Root of *Codonopsis pilosula (Franch.)Nannf.,*Root of *Astragalus membranaceus(Fisch.) Bge.var.mongholicus(Bge.)* | Y-Prepared according to Chinese pharmacopeia(2000) | Y-Spectrophotometry |
| Lu (2005) | Shenqi Fuzheng injection | Shenzhen Lizhu Group pharmaceutical Co., Ltd | Root of *Codonopsis pilosula (Franch.)Nannf.,*Root of *Astragalus membranaceus(Fisch.) Bge.var.mongholicus(Bge.)* | Y-Prepared according to Chinese pharmacopeia(2000) | Y-Spectrophotometry |
| Zhan et al. (2017) | Dazhuhongjingtian injection | Tonghua Yusheng Pharmaceutical Co., Ltd | Root of *Rhodiola crenulata (Hook. f. et Thoms.)H. Ohba* | － | － |
| Tian et al. (2017) | Dazhuhongjingtian injection | Not mentioned | Root of *Rhodiola crenulata (Hook. f. et Thoms.)H. Ohba* | － | － |
| Yang et al. (2014) | Danshenduofenyansuan injection | Shanghai Lvgu Pharmaceutical Co., Ltd | Root of *Salvia miltiorrhiza Bge.* | Y-Prepared according to Chinese pharmacopeia | Y-HPLC |
| Xu (2016) | Danshenduofenyansuan injection | Nanjing Kaiji Biological Technology Co., Ltd | Root of *Salvia miltiorrhiza Bge.* | Y-Prepared according to Chinese pharmacopeia | Y-HPLC |
| Zhang (2020) | Danshenduofenyansuan injection | Shanghai Lvgu Pharmaceutical Co., Ltd | Root of *Salvia miltiorrhiza Bge.* | Y-Prepared according to Chinese pharmacopeia | Y-HPLC |
| Zhang (2015) | Danhong injection | Shandong Danhong Pharmaceutical Co., Ltd | Root of *Salvia miltiorrhiza Bge.,*Flower of *Carthamus tinctorius L.* | Y-Prepared according to Chinese pharmacopeia(2000) | Y-Spectrophotometry |
| Wang (2012) | Danhong injection | Not mentioned | Root of *Salvia miltiorrhiza Bge.,*Flower of *Carthamus tinctorius L.* | － | － |
| Xing et al. (2009) | Dengzhanxixin injection | Yunnan Biovalley Breviscapus Pharmaceutical Co., Ltd | Herb of *Erigeron breviscapus（Vant.）Hand.-Mazz.* | Y-Prepared according to Chinese pharmacopeia(2005) | Y-HPLC |
| Teng (2016) | Gualoupi injection | Not mentioned | Pericarp of *Trichosanthes kirilowii Maxim.* | － | － |
| Ni et al. (2020) | Gualoupi injection | Shanghai First Biochemical Pharmaceutical Co., Ltd | Pericarp of *Trichosanthes kirilowii Maxim.* | Y-Prepared according to Chinese pharmacopeia(2000) | Y-Spectrophotometry |
| Han (2018) | Shenfu injection | Ya 'an Sanjiu Pharmaceutical Co., Ltd | Root of *Panax ginseng C. A. Mey.,*Subroot of *Aconitum Carmichaelii Debx* | Y-Prepared according to Chinese pharmacopeia | Y-Spectrophotometry |
| Zhou et al. (2002) | Huangqi injection | Not mentioned | Root of *Astragalus membranaceus(Fisch.) Bge.var.mongholicus(Bge.)* | － | － |
| Huang et al. (1999) | Shengmai injection | Su Zhong Pharmaceutical Factory | Root of *Panax ginseng C.A.Mey./Codonopsis pilosula (Franch.)Nannf.,*Root of *Ophiopogon japonicus （L.f） Ker-Gaw,*Fruit of *Schisandra chinensis（Turcz.）Baill.* | Y-Prepared according to Chinese pharmacopeia | Y-HPLC |
| Zhao et al. (2011) | Shengmai injection | Not mentioned | Root of *Panax ginseng C.A.Mey./Codonopsis pilosula (Franch.)Nannf.,*Root of *Ophiopogon japonicus （L.f） Ker-Gaw,*Fruit of *Schisandra chinensis（Turcz.）Baill.* | － | － |
| Wang et al. (2011) | Shuxuening injection | Shenwei Pharmaceutical Co., Ltd | Foliage of *Ginkgo biloba L.* | Y-Prepared according to Chinese pharmacopeia | Y-HPLC |
| Zhang (2015) | Shuxuetong injection | Mudanjiang Youbo Pharmaceutical Co., Ltd | Whole of *Hirudo nipponica Whitman,*Whole of *Pheretima aspergillum(E.Perrier)* | Y-Prepared according to Chinese pharmacopeia(2005) | Y-HPLC |
| Ren (2021) | Xinmailong injection | Yunnan Tengyao Pharmaceutical Co., Ltd | Extractum Xinmailong | Y-Prepared according to Chinese pharmacopeia(2005) | Y-HPLC |
| Wei et al. (2020) | Xinmailong injection | Not mentioned | Extractum Xinmailong | － | － |
| Kuang (2004) | Xuesaintong injection | Not mentioned | Root of *Panax notoginseng(Burk.)F.H.Chen* | － | － |
| Guo et al. (2012) | Shenmai injection | Hangzhou Zhengda Qingchunbao Group | Root of *Panax ginseng C. A. Mey.,* Root of *Ophiopogon japonicus （L.f） Ker-GawL* | Y-Prepared according to Chinese pharmacopeia | Y-HPLC |

***Supplementary table S4*** The included herbs and traditonal efficacy of TCMIs

| TCMI | Pharmaceutical name | Properties | Merdians | Traditional efficacy |
| --- | --- | --- | --- | --- |
| Shenfu injection | Radix Ginseng Rubra | Warm, Mild, Bitter | Bladder, Large Intestine | reviving yang |
|  | Common Monkshood Daughter Root | Extreme Hot, Pungent, Sweet | Spleen, Heart, Kidney |  |
| Shenmai injection | Panax ginseng | Minor Warm, Sweet,Slightly Bitter | Lung,Spleen,Heart | supplementing qi and nourishing yin |
|  | Ophiopogon japonicus | Minor Warm,Sweet,Slightly Bitter | Lung,Spleen,Heart |  |
| Shengmai injection | Panax ginseng | Minor Warm,Sweet,Slightly Bitter | Lung,Spleen,Heart | supplementing qi and nourishing yin |
|  | Ophiopogon japonicus | Minor Warm,Sweet,Slightly Bitter | Lung,Spleen,Heart |  |
|  | Schisandra chinensis | Warm,Sour | Lung,Heart,Kidney |  |
| Xinmailong injection | Carduus crispus | Cold,Bitter,Salty | Large Intestine,Stomach | invigorating qi and activating blood |
| Danshenduofenyan injection | Salvia miltiorrhiza | Minor cold,Bitter | Liver,Heart | invigorating the blood circlation |
| Danhong injection | Salvia miltiorrhiza | Minor cold,Bitter | Liver,Heart | invigorating the blood circulation |
|  | Carthamus tinctorius | Warm,Pungent | Liver,Heart |  |
| Dazhuhongjingtian injection | Radix et Rhizoma Rhodiolae | Minor cold,Sweet,Punkery | Lung,Liver,Kidney | invigorating the blood circulation |
| Dengzhanxixin injectin | Erigeron breviscapus | Warm, Pungent, Slightly Bitter | Heart, Liver | invigorating the blood circulation |
| Shuxuening injection | Ginkgo Folium | neutral,Sweet, Bitter | Heart, Lung | invigorating the blood circulation |
| Xuesaitong injection | Panax pseudo - ginseng var. notoginseng | Warm,Pungent,Slightly Bitter | Stomach,Liver | invigorating the blood circulation |
| Gualoupi injection | Pericarpium Trichosanthis | Cold,Sweet,Slightly Bitter | Lung,Large Intestine,Stomach | promoting the circulation of qi |
| Shuxuetong injection | Pheretima | Cold,Salty | Bladder,Spleen,Liver | invigorating the blood circulation |
|  | Pheretima | Cold,Salty | Bladder,Spleen,Liver |  |
| Shenqi Fuzheng injection | Codonopsis pilosula | Mild,Sweet | Lung,Spleen | supplementing qi |
|  | Astragalus membranaceus | Warm,Sweet | Lung,Spleen |  |
| Huangqiinjection | Astragalus membranaceus | Warm,Sweet | Lung,Spleen | supplementing qi |
| Yiqi Fumai injection | Ophiopogon japonicus | Minor Warm,Sweet,Slightly Bitter | Lung,Spleen,Heart | invigorating qi |
|  | Schisandra chinensis | Warm,Sour | Lung,Heart,Kidney |  |
|  | Radix Ginseng Rubra | Warm,Mild,Bitter | Bladder,Large Intestine |  |

| **Treatments** | **Rank of possibility %** | | | | | | | | | | | | | | |
| --- | --- | --- | --- | --- | --- | --- | --- | --- | --- | --- | --- | --- | --- | --- | --- |
|  | **1** | **2** | **3** | **4** | **5** | **6** | **7** | **8** | **9** | **10** | **11** | **12** | **13** | **14** |  |
| Shuxuetong injection | 39.24 | 0.32 | 1.32 | 5.89 | 0.01 | 0.12 | 0.31 | 0.69 | 0.93 | 22.59 | 1.34 | 19.66 | 0.02 | 7.56 |  |
| Gualoupi injection | 1.08 | 6.51 | 11.85 | 17.44 | 1.52 | 3.1 | 3.63 | 3.96 | 5.66 | 14.67 | 4.21 | 13.15 | 0.32 | 12.9 |  |
| Danshenduofenyansuan injection | 0 | 15.4 | 12.64 | 8.01 | 15.21 | 10.13 | 8.59 | 5.74 | 7.58 | 2.53 | 3.86 | 2.97 | 1.56 | 5.78 |  |
| Shenqi Fuzheng injection | 0 | 14.33 | 10.67 | 6.63 | 17.96 | 11.86 | 9.8 | 6.08 | 8.05 | 1.74 | 3.75 | 2.13 | 2.17 | 4.84 |  |
| Danhong injection | 0 | 14.86 | 14.76 | 10.38 | 9.77 | 8.21 | 7.65 | 5.67 | 7.73 | 4.22 | 3.92 | 4.42 | 1.05 | 7.36 |  |
| Xinmailong injection | 0 | 12.04 | 8.86 | 5.97 | 18.78 | 13.23 | 10.89 | 6.9 | 8.29 | 1.18 | 4.23 | 1.84 | 2.96 | 4.84 |  |
| Shenfu injection | 0 | 9.72 | 7.68 | 5.17 | 15.81 | 14.9 | 12.15 | 8.14 | 9.68 | 0.82 | 5 | 1.36 | 4.68 | 4.8 |  |
| Shenmai injection | 0 | 1.71 | 1.8 | 2.35 | 1.3 | 5.61 | 9.15 | 16.38 | 11.03 | 0.2 | 15.61 | 0.83 | 28.22 | 6.09 |  |
| Shengmai injection | 0 | 4.13 | 3.82 | 3.68 | 4.7 | 10.48 | 13.02 | 14.79 | 13.07 | 0.33 | 9.87 | 0.54 | 15.28 | 6.09 |  |
| Xuesaitong injection | 48.89 | 0 | 0.05 | 1.72 | 0 | 0 | 0.01 | 0.12 | 0.15 | 18 | 0.73 | 23.26 | 0 | 5.99 |  |
| Yiqi Fumai injection | 0 | 7.18 | 5.74 | 4.79 | 9.95 | 14.42 | 13.54 | 10.88 | 11.42 | 0.58 | 6.89 | 1.11 | 7.95 | 7.08 |  |
| Huangqi injection | 10.72 | 2.24 | 5.18 | 12.91 | 0.28 | 0.92 | 1.47 | 1.97 | 3.1 | 25.31 | 2.75 | 20.87 | 0.12 | 12.15 |  |
| Dengzhanxixin injection | 0.07 | 11.16 | 15.18 | 14.34 | 4.51 | 5.46 | 5.93 | 5.48 | 7.38 | 7.8 | 4.08 | 7.68 | 0.62 | 10.33 |  |
| CT | 0 | 0.41 | 0.45 | 0.73 | 0.19 | 1.56 | 3.86 | 13.22 | 5.93 | 0.05 | 33.75 | 0.18 | 35.05 | 4.64 |  |

***Supplementary table S5*** Bayesian ranking results of network meta-analysis (Total effective rate).The number in each cell represents the posterior probability of the row-defining treatment being ranked at the column-defining position.

| **Treatments** | **Rank of possibility %** | | | | | | | | | | | | |
| --- | --- | --- | --- | --- | --- | --- | --- | --- | --- | --- | --- | --- | --- |
|  | **1** | **2** | **3** | **4** | **5** | **6** | **7** | **8** | **9** | **10** | **11** | **12** | **13** |
| Shenmai injection | 0 | 5.23 | 20.37 | 10.95 | 16.61 | 4.03 | 12.49 | 3.36 | 9.63 | 1.47 | 3.56 | 3.51 | 8.78 |
| Yiqi Fumai injection | 0 | 17.62 | 6.47 | 10.16 | 7.34 | 11.44 | 7.64 | 4.6 | 14.04 | 2.52 | 4.9 | 4.91 | 8.37 |
| Dazhuhongjingtian injection | 0.06 | 18.49 | 1.9 | 9.08 | 3.75 | 16.76 | 5.78 | 6.22 | 12.78 | 3.93 | 6.46 | 6.63 | 8.18 |
| Shengmai injection | 0 | 11.57 | 11.85 | 11.13 | 11.16 | 7.73 | 9.73 | 4.07 | 13.13 | 1.98 | 4.36 | 4.4 | 8.88 |
| Danshenduofenyansuan injection | 4.83 | 7.63 | 0.48 | 8.4 | 1.85 | 13.69 | 4.92 | 10.38 | 8.79 | 7.96 | 10.76 | 10.96 | 9.35 |
| Shenfu injection | 0 | 1.54 | 28.46 | 9.51 | 22.17 | 1.52 | 14.71 | 2.54 | 5.47 | 0.99 | 2.67 | 2.59 | 7.82 |
| Shuxuening injection | 11.44 | 0 | 0 | 1.53 | 0.06 | 0.23 | 1.34 | 17.14 | 0.28 | 33.1 | 15.55 | 14.82 | 4.51 |
| Xinmailong injection | 0 | 20.23 | 3.51 | 9.34 | 5.16 | 14.56 | 6.4 | 5.19 | 13.69 | 3.03 | 5.42 | 5.56 | 7.93 |
| Gualoupi injection | 31.8 | 0.1 | 0.01 | 2.62 | 0.17 | 1 | 1.9 | 13.5 | 0.9 | 16.77 | 12.94 | 12.9 | 5.39 |
| Dengzhanxixin injection | 33.66 | 0.84 | 0.05 | 4.07 | 0.44 | 3.47 | 2.57 | 11.49 | 2.42 | 11.9 | 11.43 | 11.48 | 6.21 |
| Shuxuetong injection | 0.69 | 13.45 | 1.03 | 9.08 | 2.73 | 16.87 | 5.49 | 8.05 | 11.36 | 5.46 | 8.25 | 8.44 | 9.09 |
| Danhong injection | 17.53 | 3.07 | 0.19 | 6.44 | 1.04 | 8.35 | 3.77 | 11.74 | 5.39 | 10.33 | 11.93 | 12.08 | 8.13 |
| CT | 0 | 0.22 | 25.67 | 7.69 | 27.53 | 0.35 | 23.27 | 1.73 | 2.13 | 0.55 | 1.79 | 1.72 | 7.37 |

***Supplementary table S6*** Bayesian ranking results of network meta-analysis (LVEF).The number in each cell represents the posterior probability of the row-defining treatment being ranked at the column-defining position.

| **Treatments** | **Rank of possibility %** | | | |
| --- | --- | --- | --- | --- |
|  | **1** | **2** | **3** | **4** |
| Xinmailong injection | 0 | 0.01 | 99.99 | 0 |
| Shenfu injection | 99.92 | 0 | 0 | 0.08 |
| CT | 0 | 99.99 | 0.01 | 0 |
| Shenmai injection | 0.08 | 0 | 0 | 99.92 |

***Supplementary table S7*** Bayesian ranking results of network meta-analysis (NT-proBNP).The number in each cell represents the posterior probability of the row-defining treatment being ranked at the column-defining position.

| **Treatments** | **Rank of possibility %** | | | | | | | | | |
| --- | --- | --- | --- | --- | --- | --- | --- | --- | --- | --- |
|  | **1** | **2** | **3** | **4** | **5** | **6** | **7** | **8** | **9** | **10** |
| Shenqi Fuzheng injection | 0 | 0.17 | 20.78 | 0 | 0 | 0 | 42.55 | 0.04 | 36.46 | 0 |
| Shuxuetong injection | 1.58 | 0 | 0.16 | 0 | 0 | 0 | 0 | 98.25 | 0 | 0 |
| Shenfu injection | 0 | 0 | 14.78 | 0.44 | 0.02 | 0 | 26.35 | 0.01 | 58.4 | 0 |
| Xinmailong injection | 0 | 0 | 0.23 | 91.31 | 8.32 | 0 | 0.13 | 0 | 0 | 0 |
| Gualoupi injection | 94.24 | 0.64 | 4.11 | 0 | 0 | 0 | 0.12 | 0.89 | 0 | 0 |
| Dazhuhongjingtian injection | 0 | 14.63 | 50.55 | 0 | 0 | 0 | 29.45 | 0.24 | 5.13 | 0 |
| Yiqi Fumai injection | 0 | 0 | 0.07 | 8.25 | 91.64 | 0 | 0.02 | 0 | 0 | 0.02 |
| CT | 0 | 0 | 0 | 0 | 0 | 84.46 | 0 | 0 | 0 | 15.53 |
| Shenmai injection | 0 | 0 | 0 | 0 | 0.01 | 15.53 | 0 | 0 | 0 | 84.45 |
| Danhong injection | 4.18 | 84.56 | 9.32 | 0 | 0 | 0 | 1.37 | 0.57 | 0 | 0 |

***Supplementary table S8*** Bayesian ranking results of network meta-analysis (BNP).The number in each cell represents the posterior probability of the row-defining treatment being ranked at the column-defining position.

| **Treatments** | **Rank of possibility %** | | | | | |
| --- | --- | --- | --- | --- | --- | --- |
|  | **1** | **2** | **3** | **4** | **5** | **6** |
| Danhong injection | 99.24 | 0 | 0.05 | 0 | 0.71 | 0 |
| Shenmai injection | 2 | 45.57 | 50.9 | 0.21 | 1.83 | 1.48 |
| Yiqi Fumai injection | 0 | 49.91 | 34.75 | 5.49 | 9.8 | 0.05 |
| Dazhuhongjingtian injection | 0.76 | 0.36 | 4.73 | 46.52 | 47.62 | 0 |
| Xinmailong injection | 4 | 4.03 | 8.16 | 47.78 | 40.03 | 0 |
| CT | 0 | 0.12 | 1.41 | 0 | 0 | 98.47 |

***Supplementary table S9*** Bayesian ranking results of network meta-analysis (6MWT).The number in each cell represents the posterior probability of the row-defining treatment being ranked at the column-defining position.

| Model | Total effective rate | LVEF | NT-proBNP | BNP | 6WMT |
| --- | --- | --- | --- | --- | --- |
| Consistency | 389.1 | 242.1 | 1991.0 | 1090.0 | 106.1 |
| Inconsistency | 389 | 260.9 | 1992.0 | 1091.0 | 106.1 |

***Supplementary table S10*** Comparisons of the fit of consistency and inconsistency models using deviance information criteria (DIC).

The DIC is a Bayesian model evaluation criterion that measures model fit adjusted with complexity of the model; smaller DIC values correspond to more preferable models. (Reference: Spiegelhalter, D.J., Best, N.G., Carlin, B.P., Van der Linde, A. Bayesian measures of model complexity and fit. Journal of the Royal Statistical Society Series B (Statistical Methodology) 2002; 64(4):583-639).


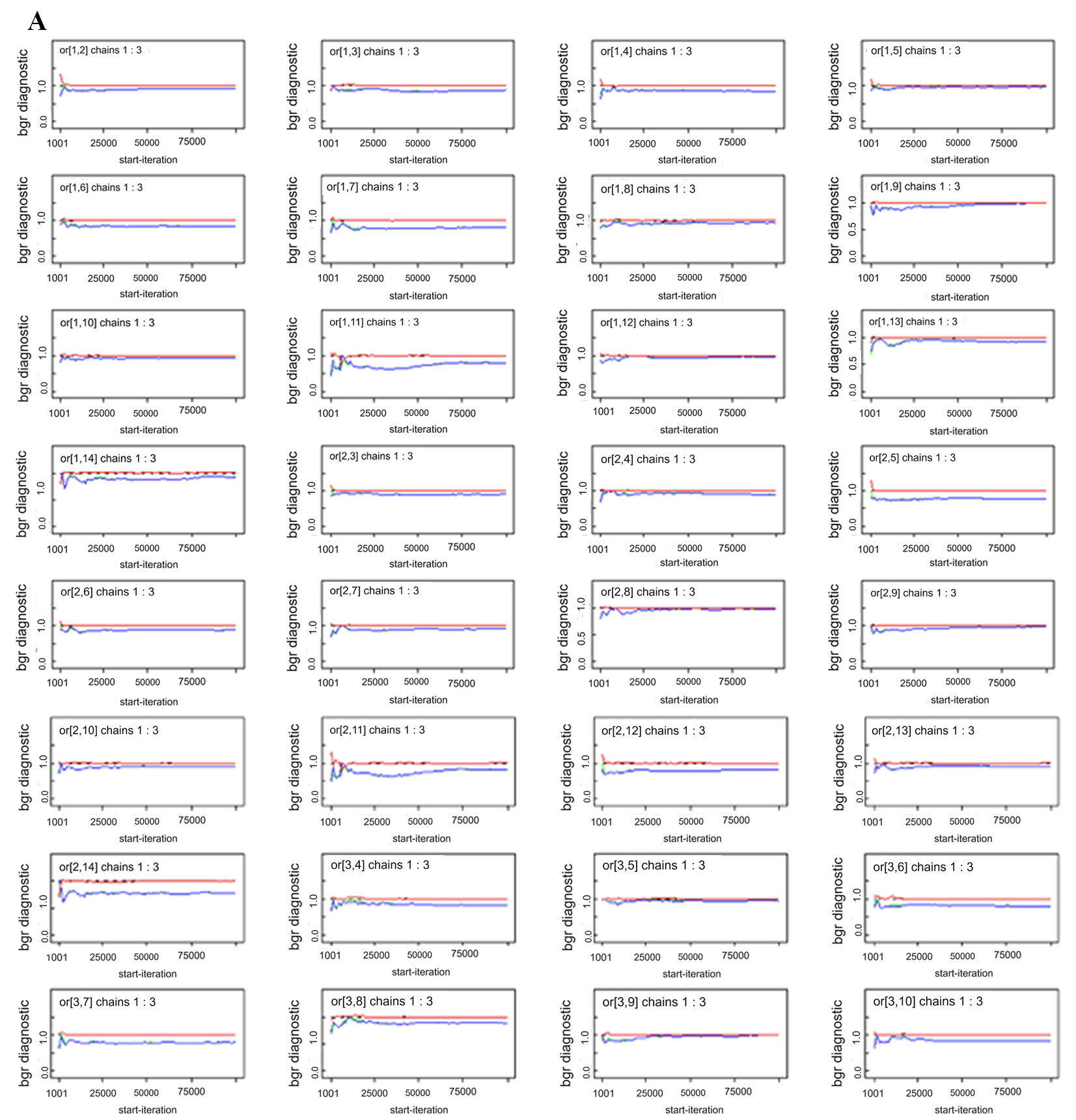


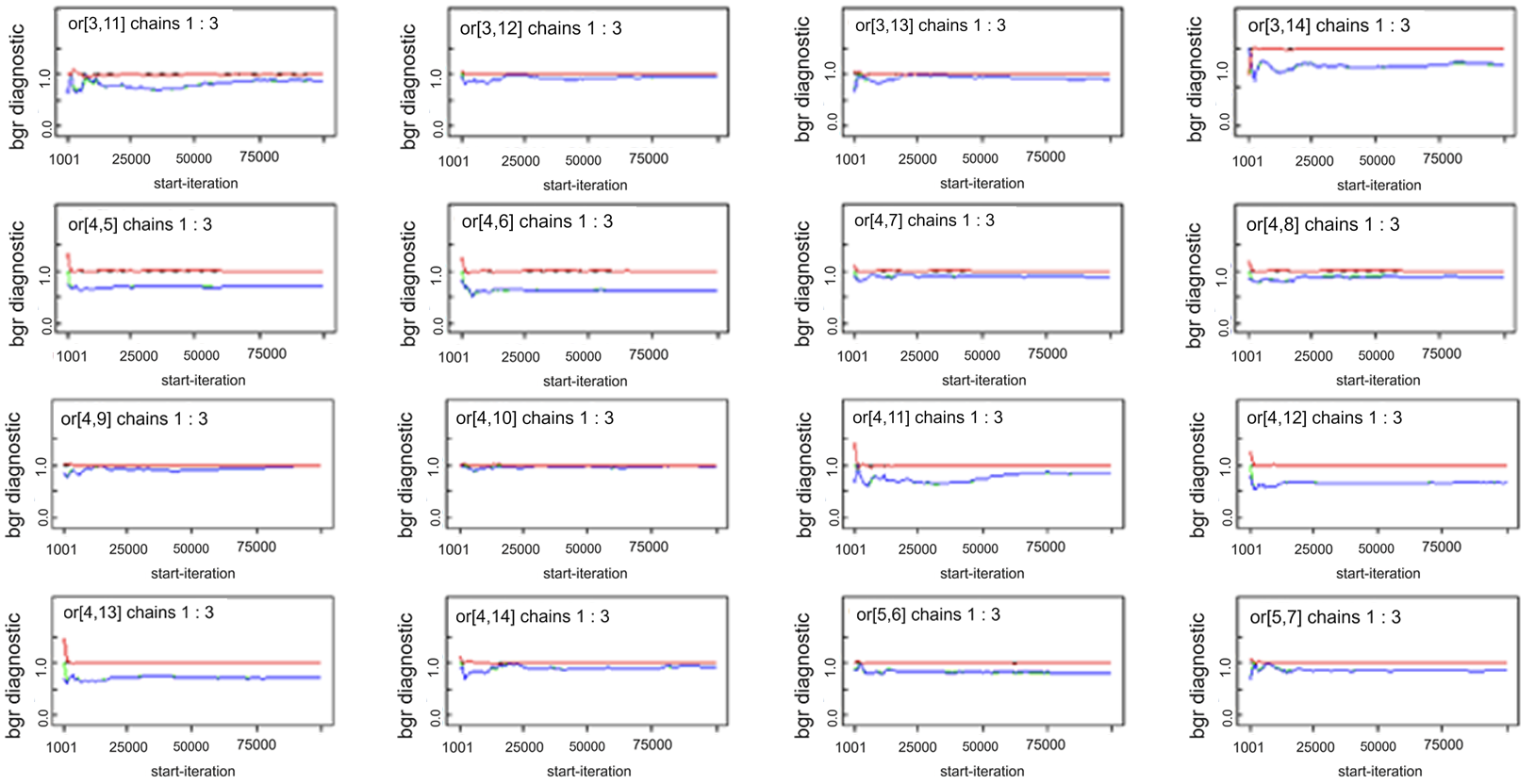


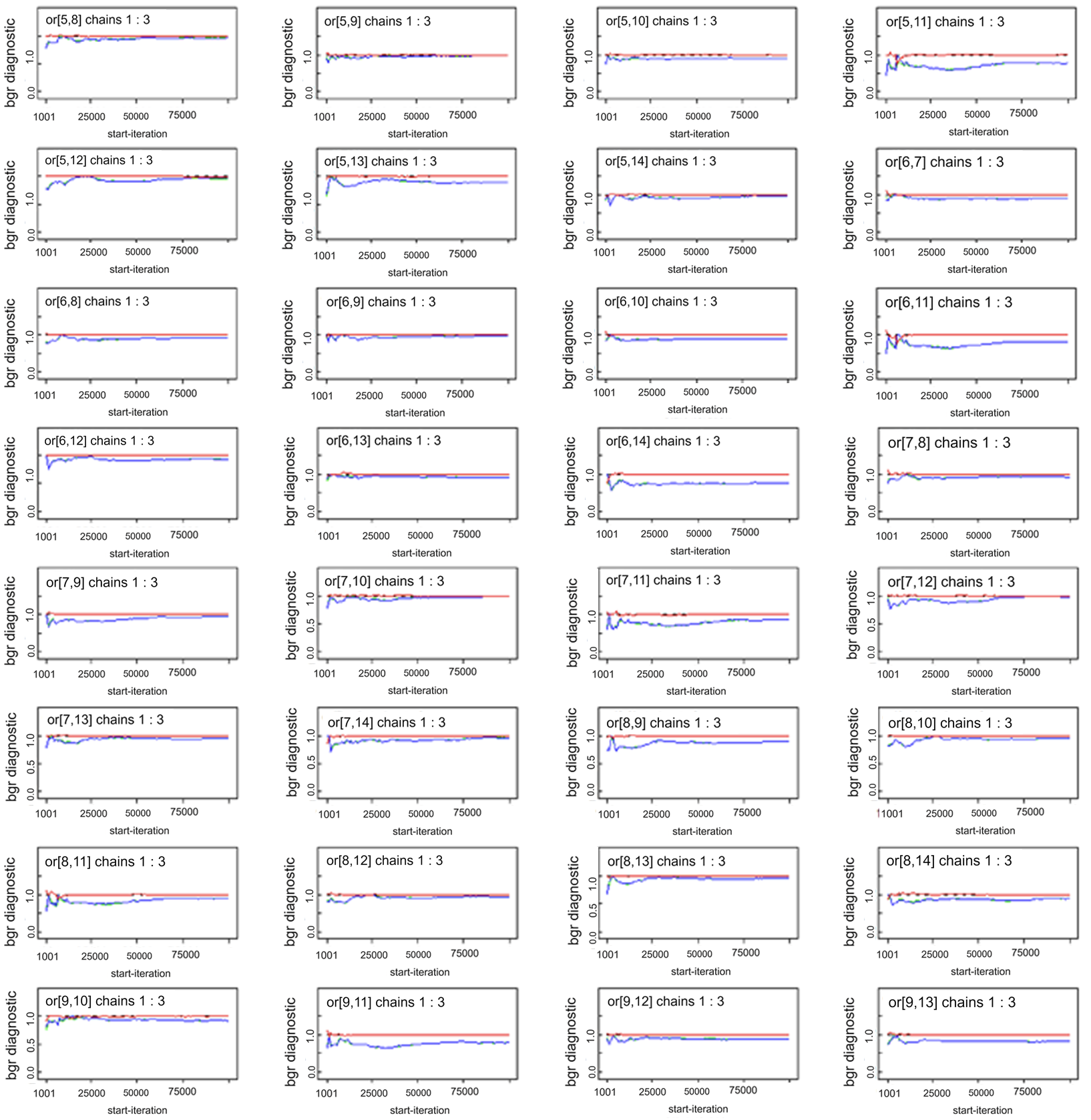


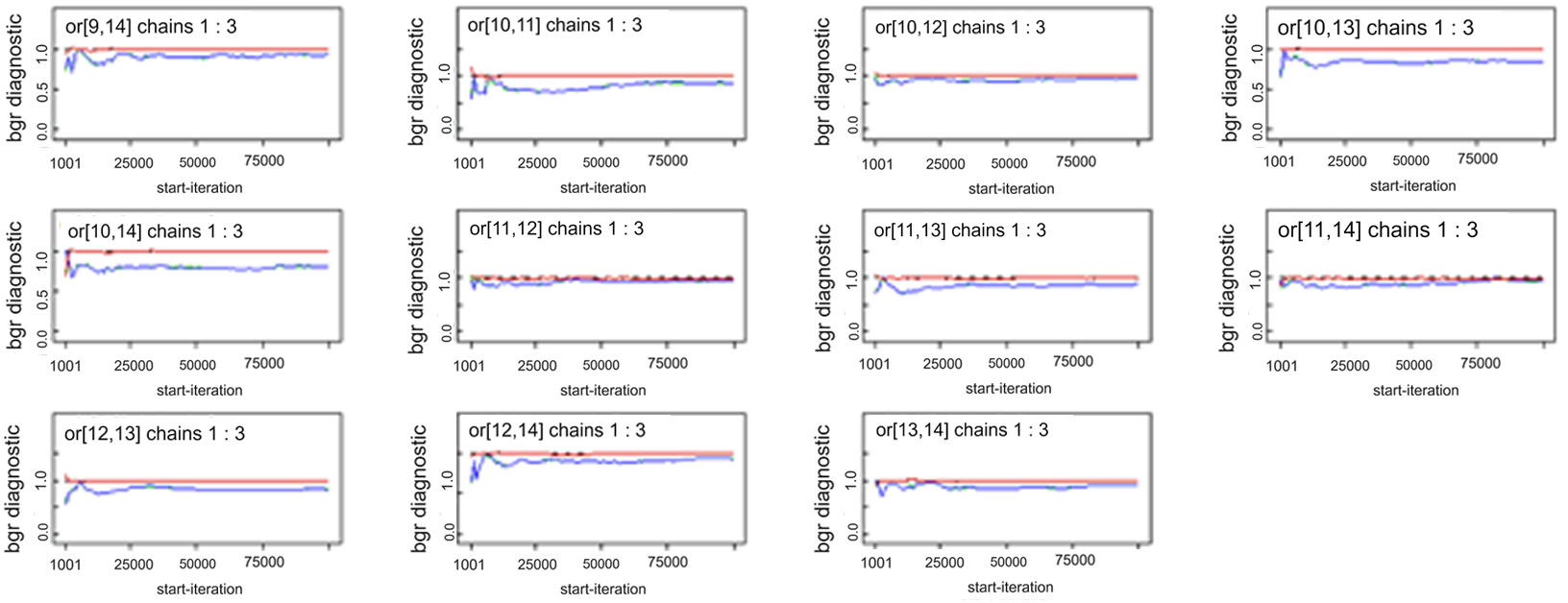


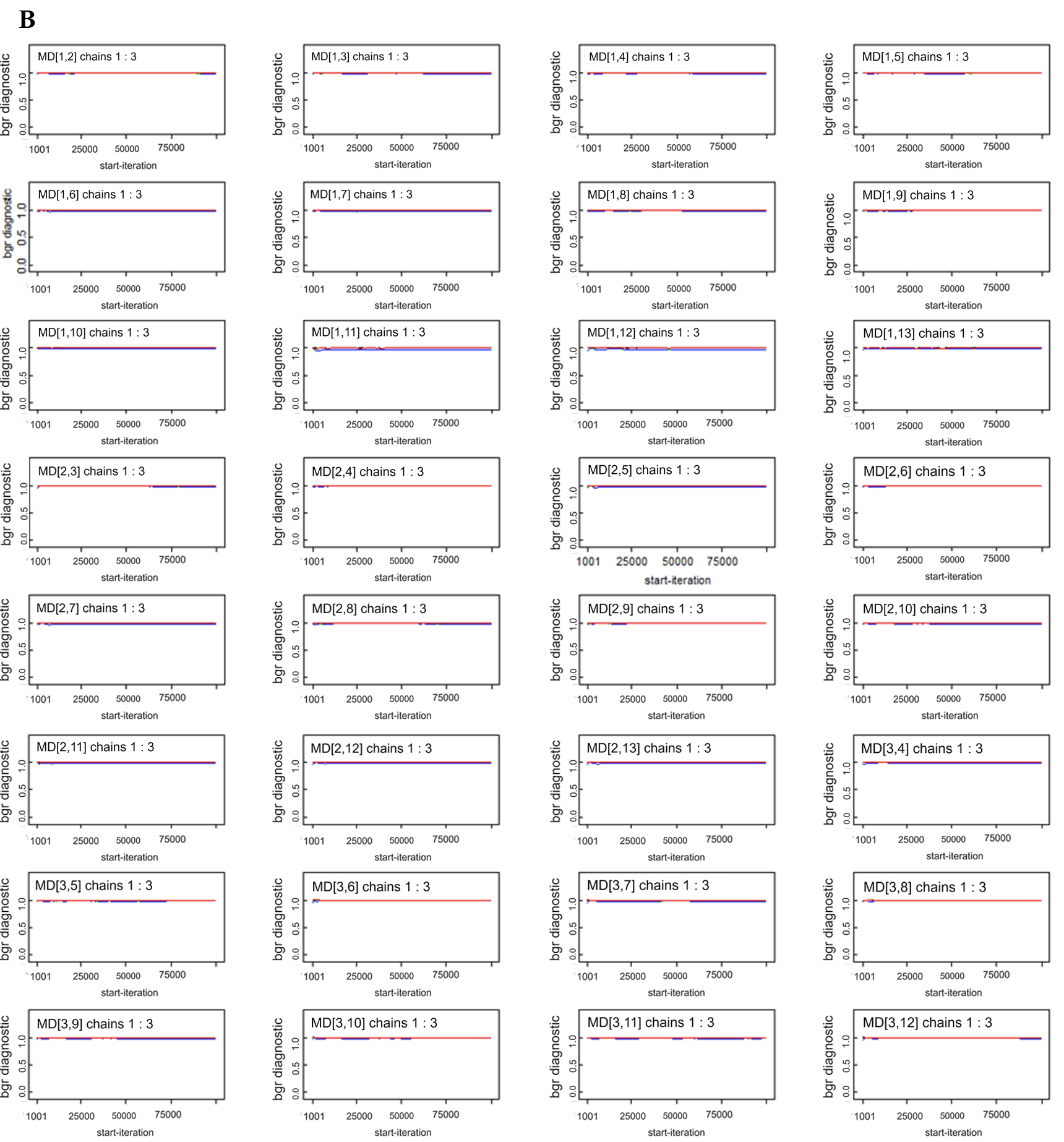


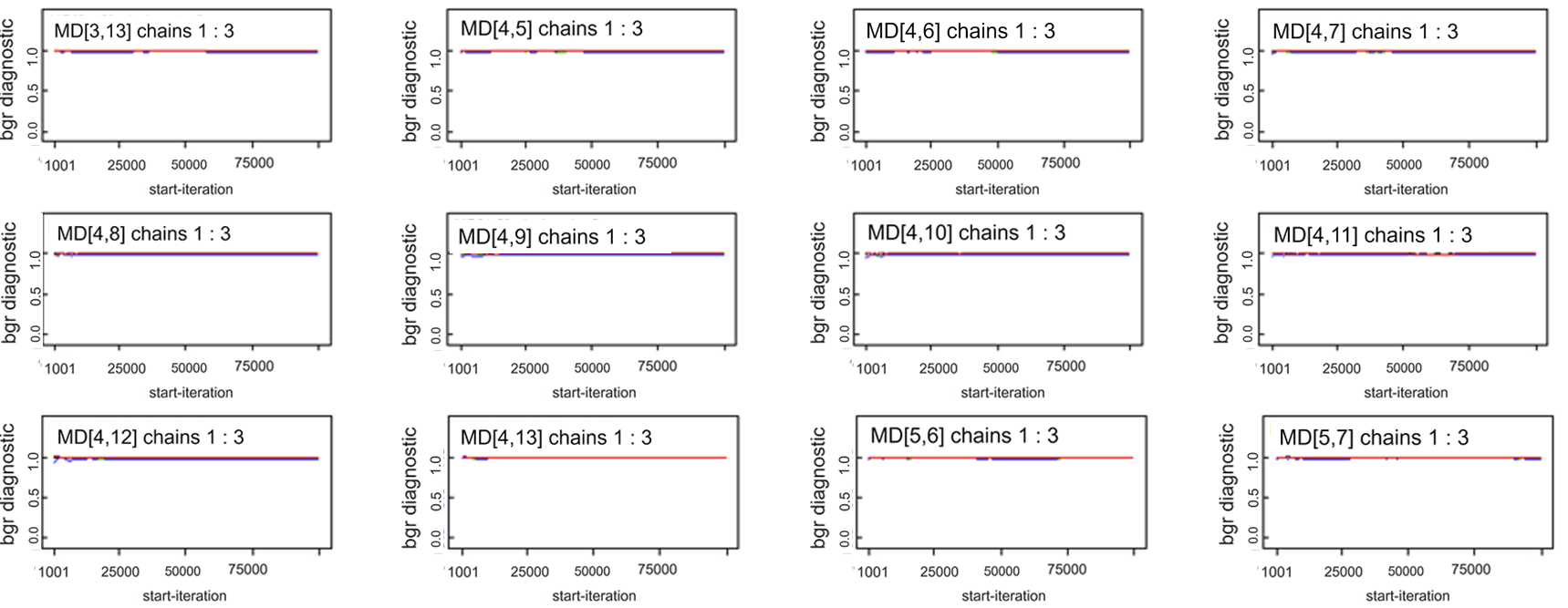


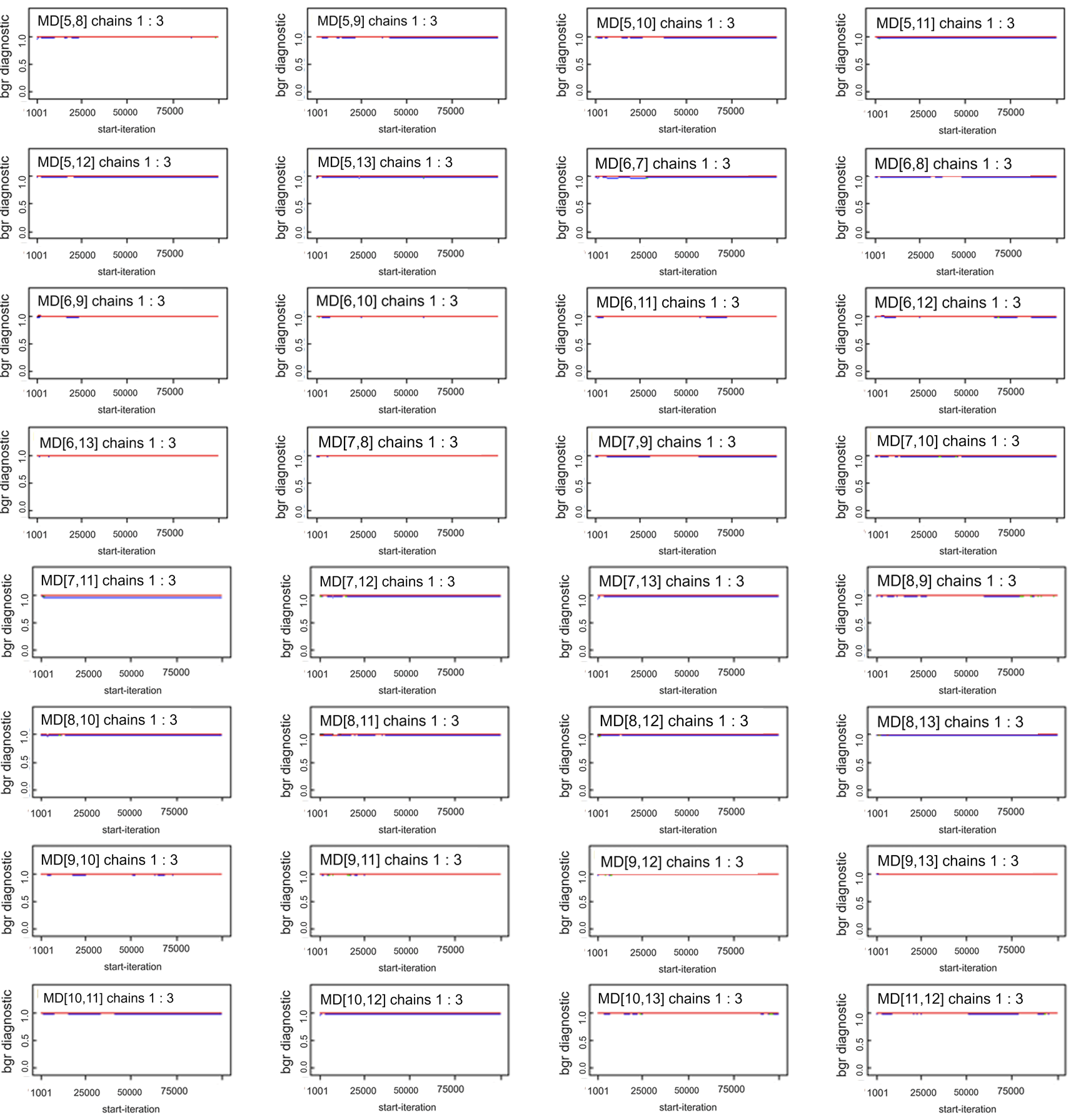


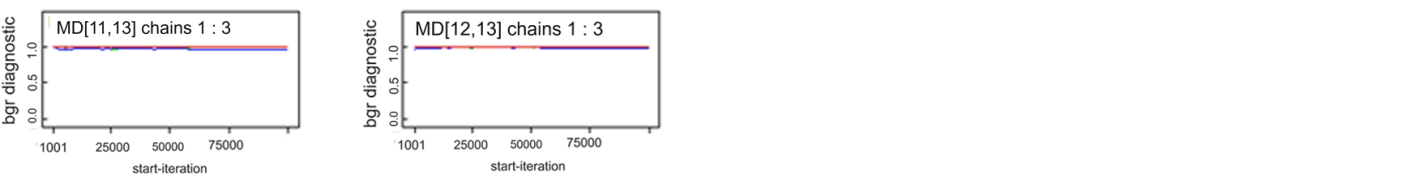


**C**


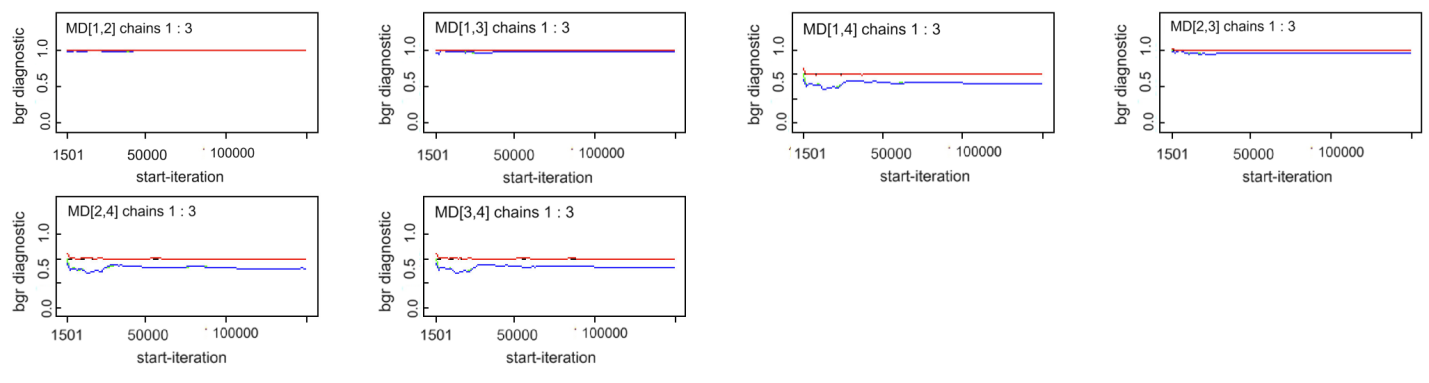


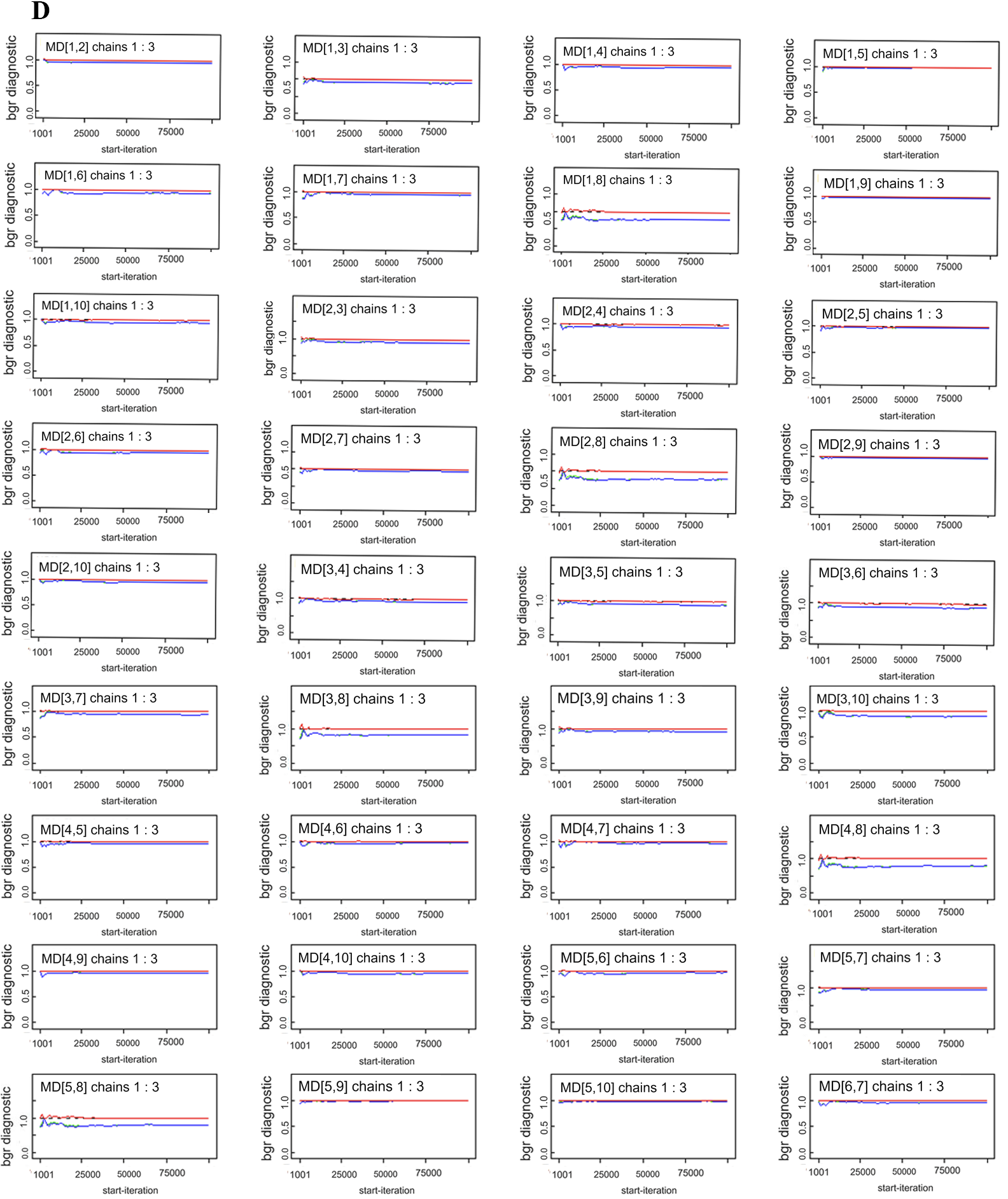


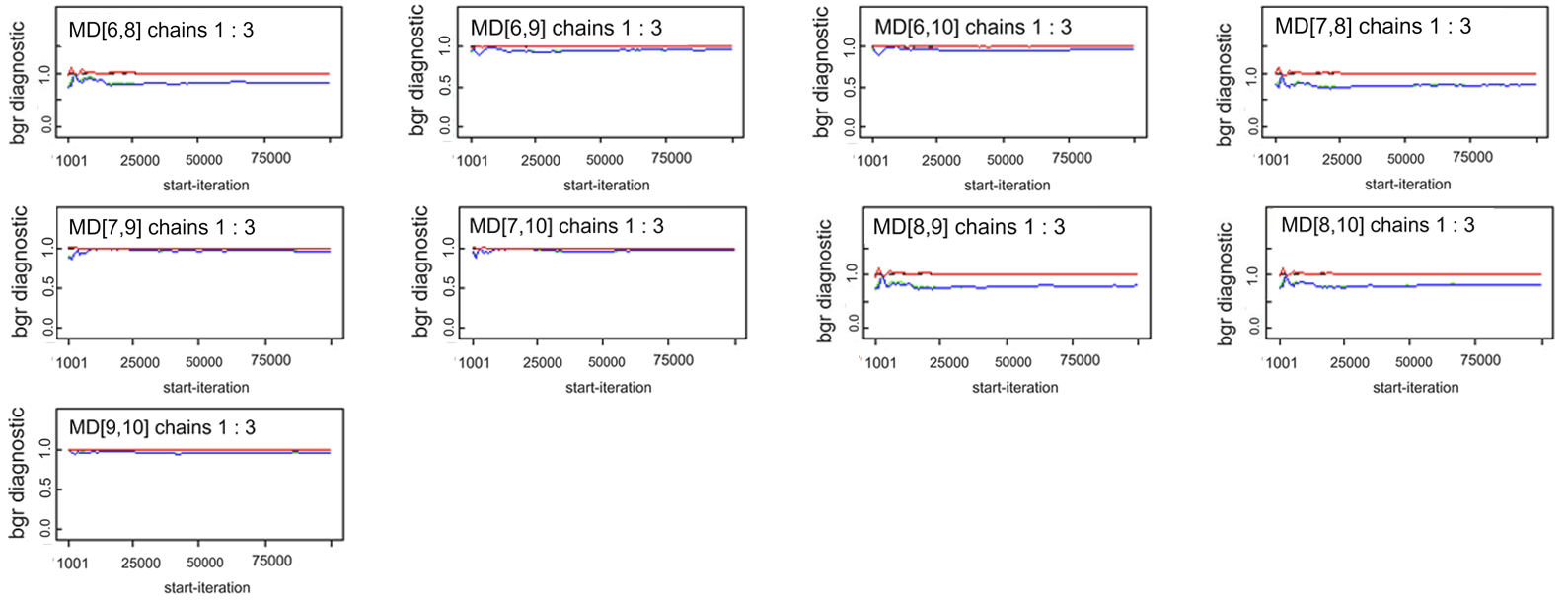


**E**


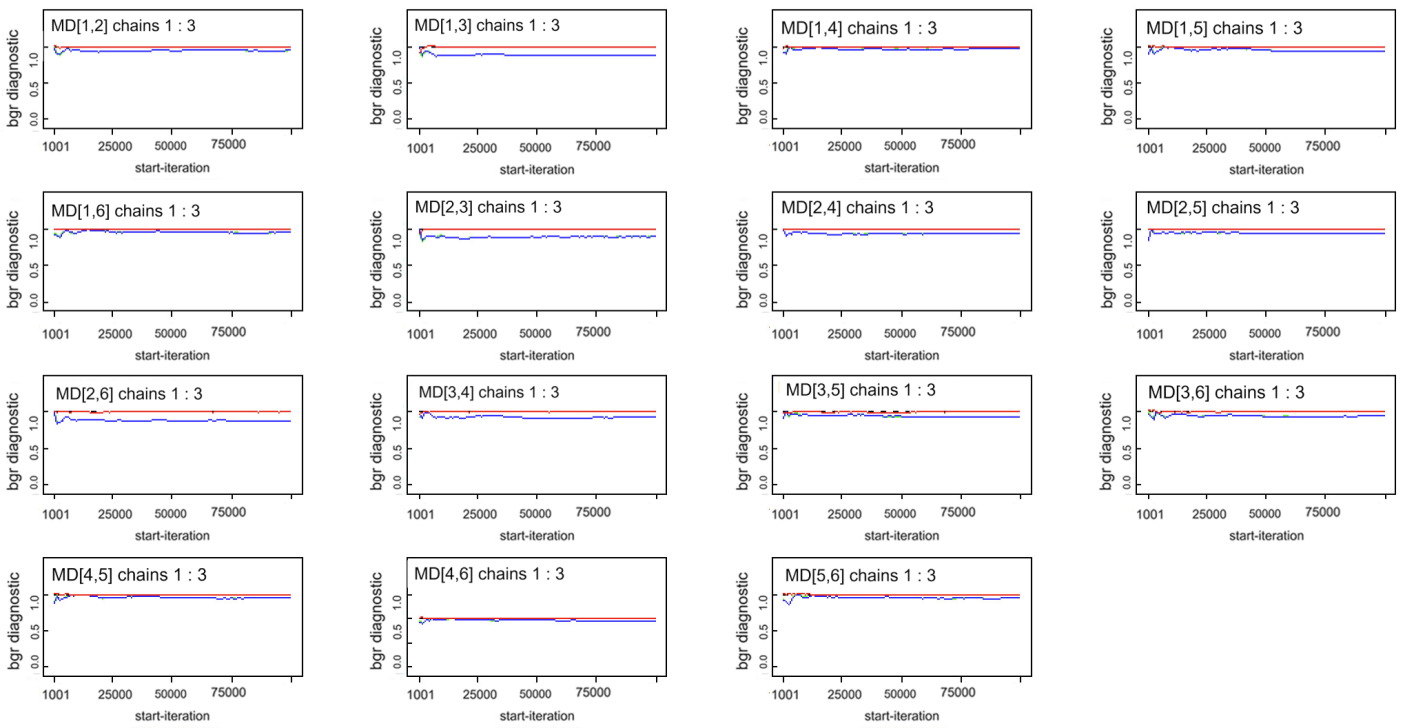


***Supplementary figure S1* Convergence of the three chains established by inspection of Brooks-Gelman-Rubin diagnostic of total effective rate (A), LVEF (B), NT-proBNP (C), BNP (D) and 6MWT (E).**


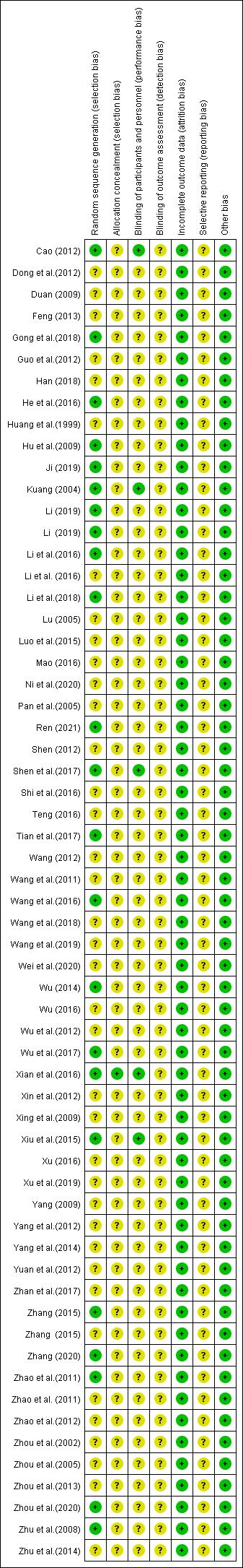


***Supplementary figure S2*** Summary of results from assessment of studies using the Cochrane risk of bias tool.


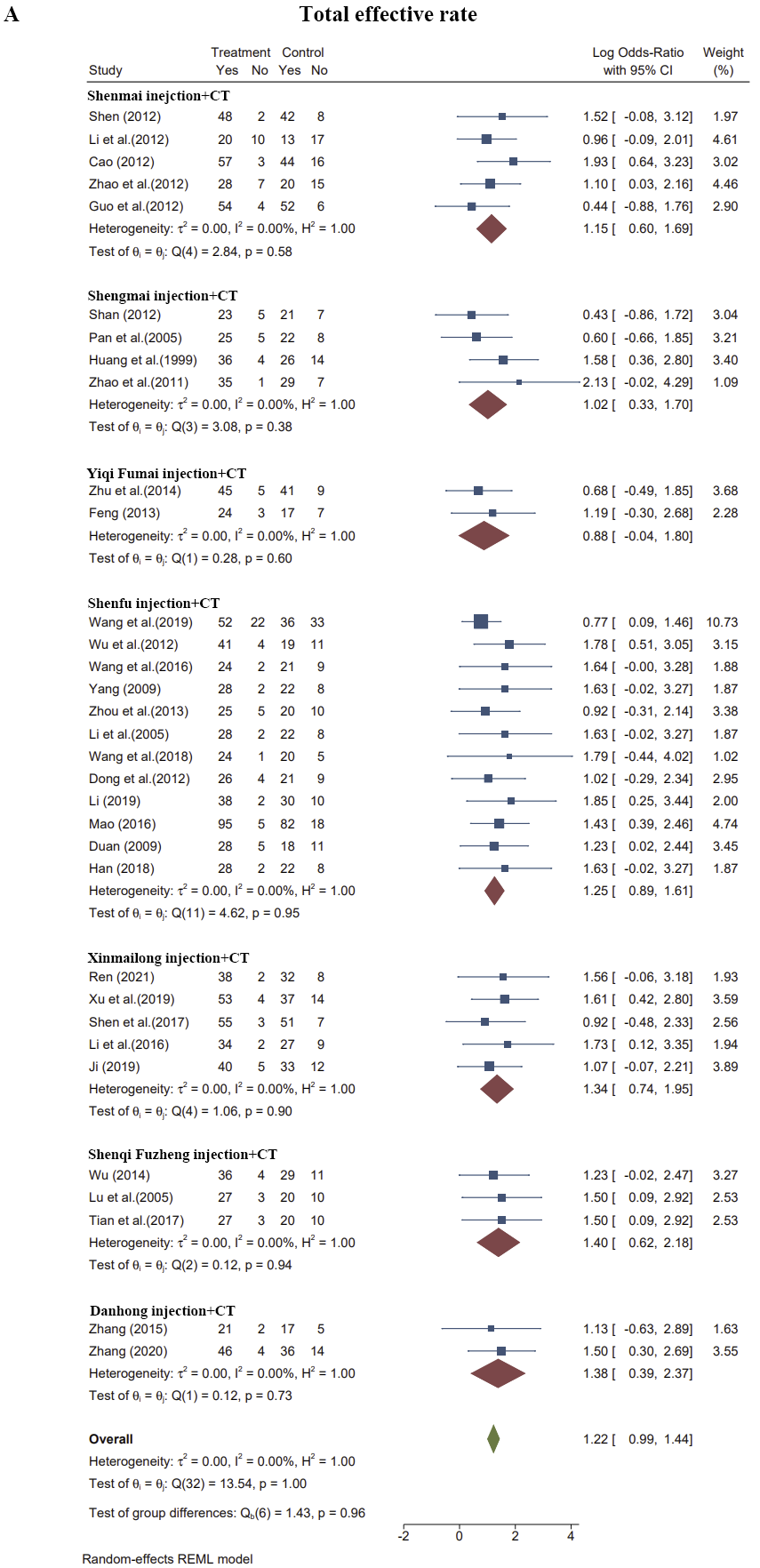

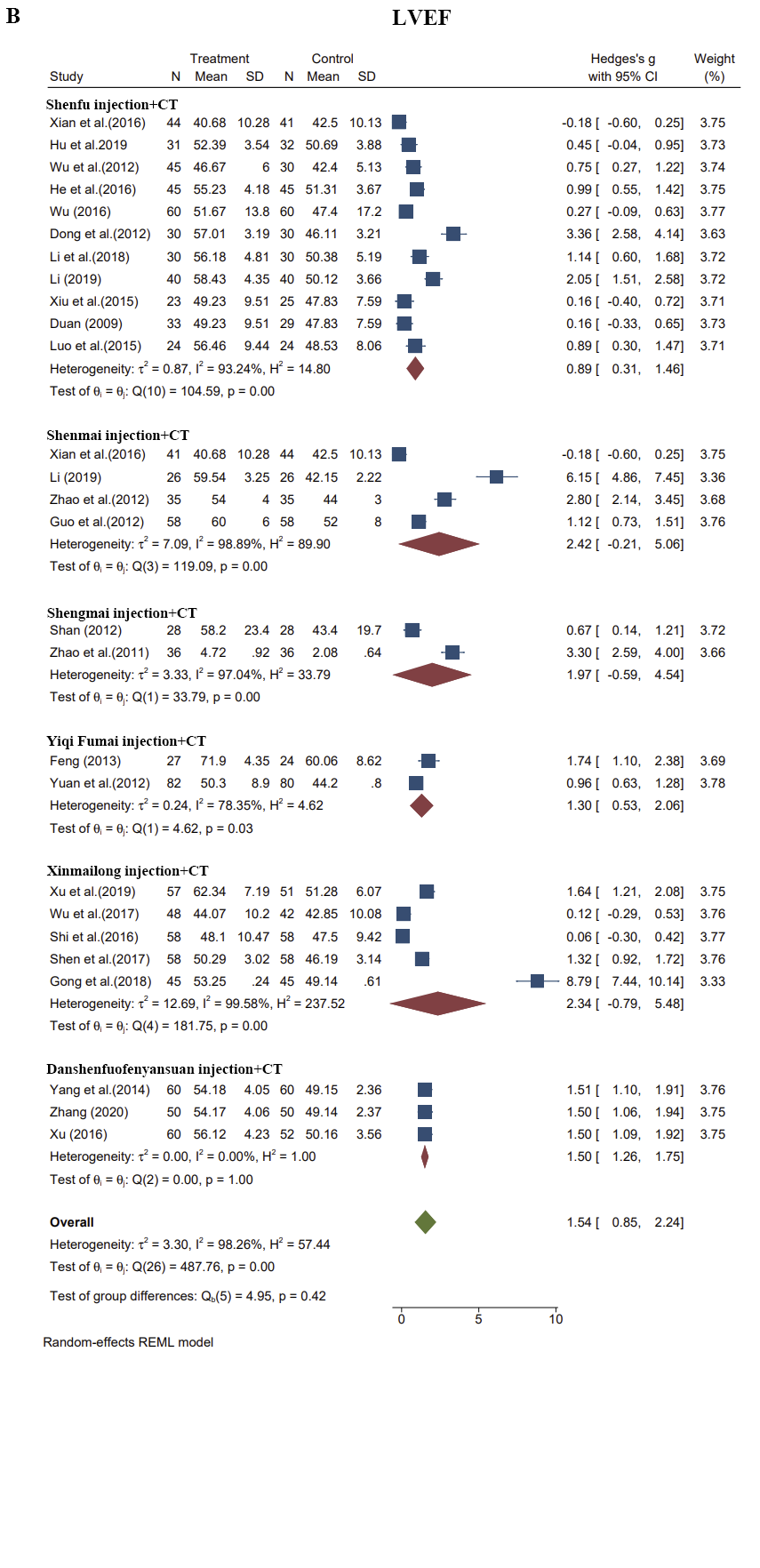


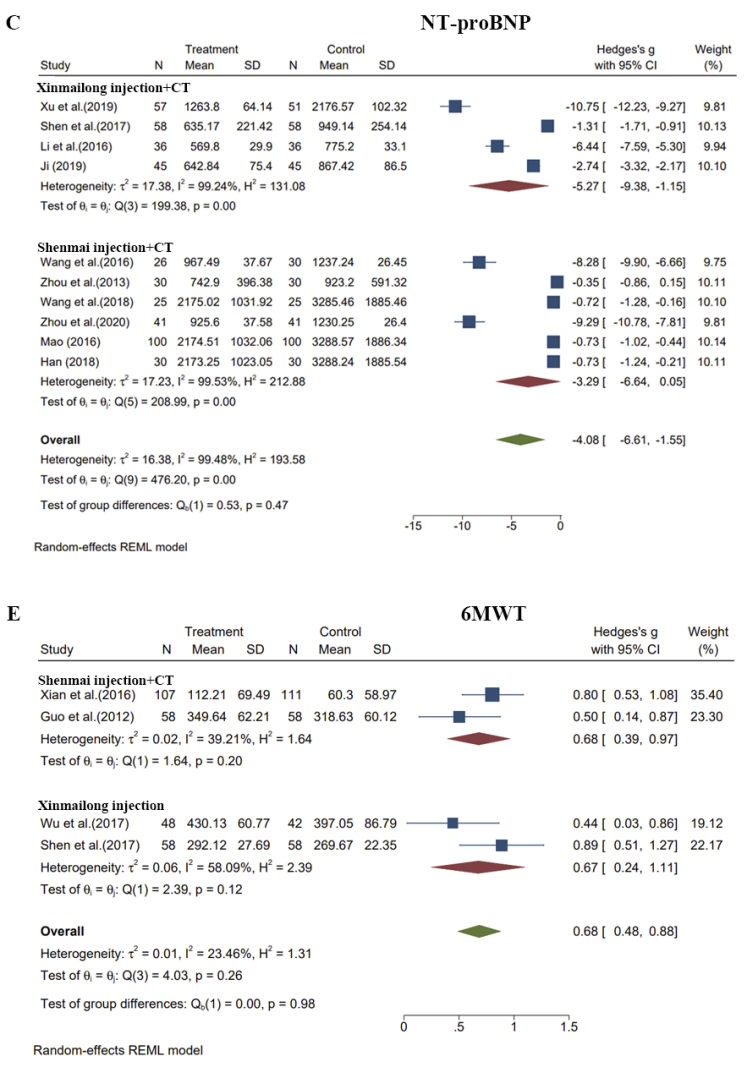

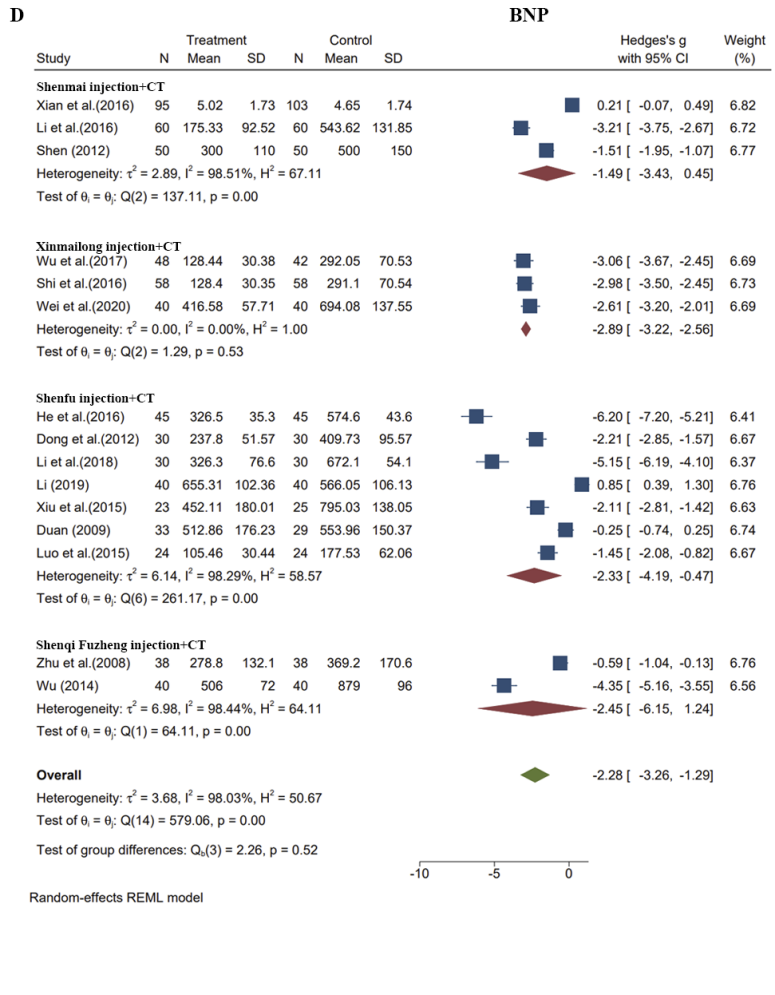


***Supplementary figure S3*** Forest plots depicting results of head-to-head comparisons according to frequentist pairwise meta-analyses on different outcomes in CHD-HF. Results of heterogeneity assessments are adherently presented. Comparisons assessed in only one trial were not plotted.


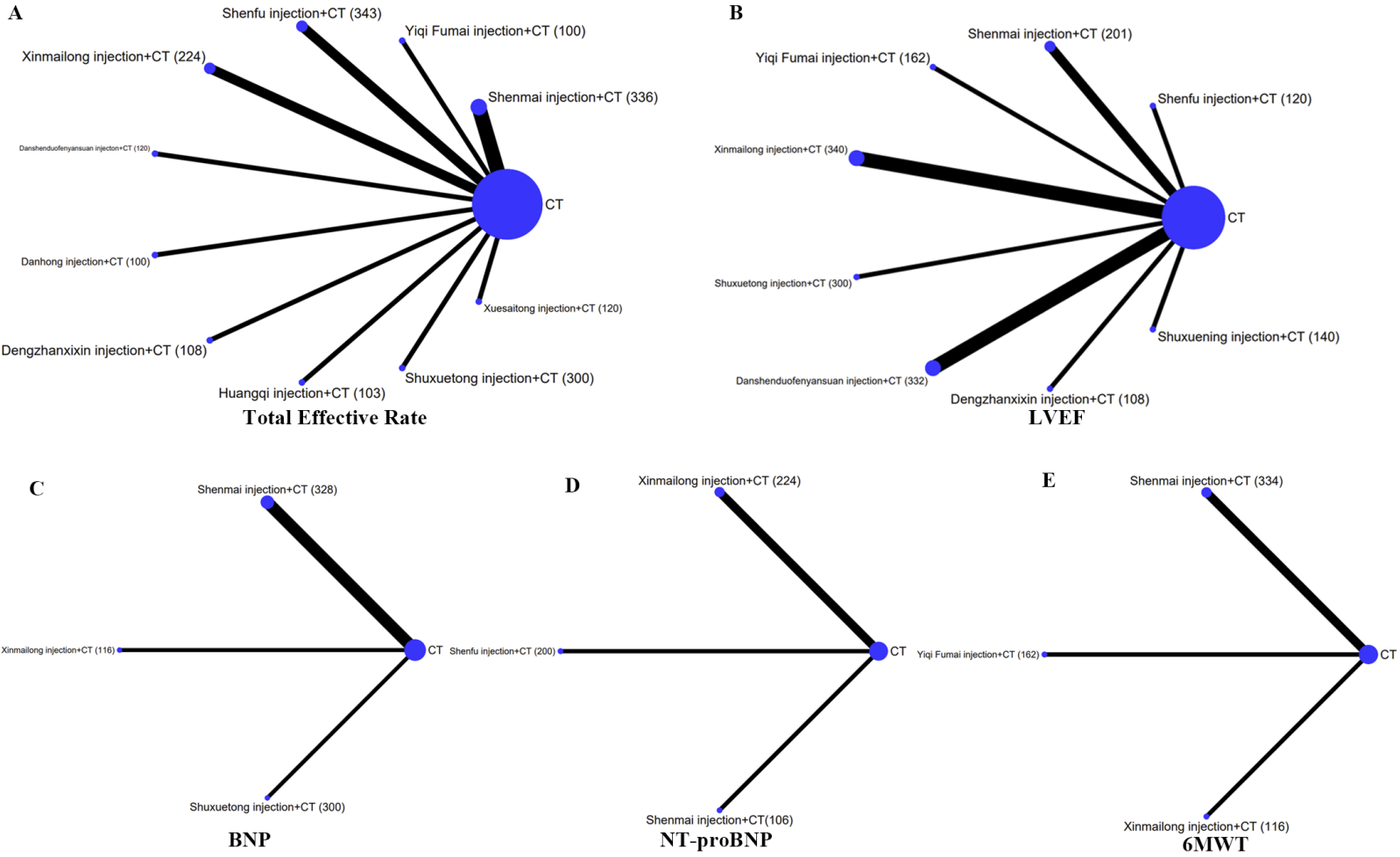


***Supplementary figure S4*** Network diagrams for the sensitive analysis (case number ≥100). The node size is proportional to the total number of patients receiving a treatment (in brackets). Each line represents a type of head-to-head comparison. The width of lines is proportional to the number of trials comparing the connected treatments.


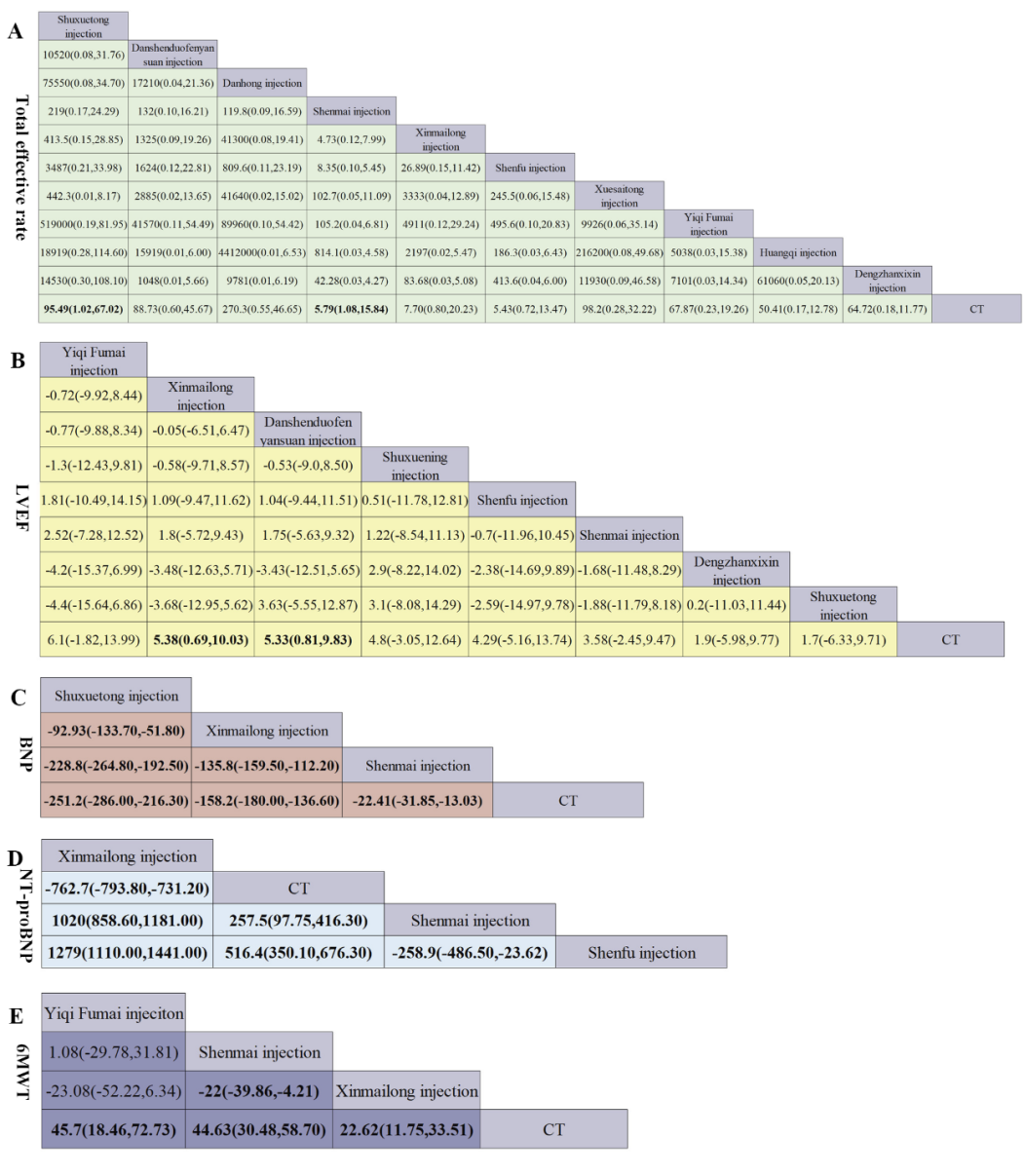


***Supplementary figure S5*** Pooled estimates of the sensitive analysis (case number ≥100). (A) Pooled odd ratios (95% credible intervals) for the total effective rate. (B) Pooled odd ratios (95% credible intervals) for LVEF. (C) Pooled odd ratios (95% credible intervals) for NT-proBNP. (D) Pooled odd ratios (95% credible intervals) for BNP. (E) Pooled odd ratios (95% credible intervals) for 6MWT. Data in each cell are hazard or odds ratios (95% credible intervals) for the comparison of row-defining treatment versus column-defining treatment. Significant results are in bold. All the TCMIs based on CT.

***Supplemantary references:***

1. Yang, F.W., Zou, J.H., Ge, L. et al. (2018). Chinese herbal injections for heart failure: A protocol for systematic review and network meta-analyses. *Medicine (Baltimore)*. 97(8), e9973. doi:10.1097/MD.0000000000009973
2. Liu, C.X., Hou, Y.Z., Wang, X.L. et al. (2015). Clinical assessment of Shenfu injection loading in the treatment of patients with exacerbation of chronic heart failure due to coronary heart disease: study protocol for a randomized controlled trial.*Trials*.16(undefined),222.

doi:10.1186/s13063-015-0729-7

1. Guo, B.B., Yang, T., Nan, J.N. et al. (2021). Efficacy and safety of Shenfu injection combined with sodium nitroprusside in the treatment of chronic heart failure in patients with coronary heart disease: A protocol of randomized controlled trial. *Medicine (Baltimore)*. 100(7), e24414. doi:10.1097/MD.0000000000024414
2. Gao, Y.B., Gao, Y., Zhu, R. et al. (2021). Shenfu injection combined with furosemide in the treatment of chronic heart failure in patients with coronary heart disease: A protocol of randomized controlled trial. *Medicine (Baltimore)*. 100(3), e24113.

doi:10.1097/MD.0000000000024113

1. Effect of Shenfu injection on hemodynamics and clinical efficacy in patients with cardiogenic shock in acute decompensation of chronic heart failure due to coronary heart disease: a multicenter, randomized, double-blinded, parallel placebo-controlled trial, ChiCTR, 2019.
2. Yan, Y.C., Han, S.M. (2014). Danhong injection improves 47 cases of coronary heart disease and heart failure in the elderly. *Shanxi. J. Tradit. Chin. Med.* 000 (002): 132-133.
3. Zhang,Z.M.(2003). Shenfu Injection in the treatment of 30 cases of senile coronary heart disease and chronic congestive heart failure. *Fujian. J. Tradit. Chin. Med*. 34,(4): 24-24.
4. Jia, L. (2010). Safflower injection in the treatment of 108 elderly patients with coronary heart disease and heart failure. *Shanxi. J. Tradit. Chin. Med.* 31 (002): 139-140.
5. Huang, M.X., Zhou, Y.G., Xie, J.Z., et al. (2019). Effect of Shenfu Injection on senile coronary heart disease and chronic heart failure. *Chron. Pathematol. J.* 20 (09): 126-127 +130.
6. Peng, Q. (2011). Clinical study on Salvia miltiorrhiza combined with Astragalus Injection in the treatment of senile coronary heart disease and heart failure. *Neimenggu. J. Tradit. Chin. Med*. 30,(09): 16-17.
7. Jin, J., Du, Y.H., Zheng, J. (2017). Analysis of clinical effect of Danhong Injection on heart failure in elderly patients with coronary heart disease. *Sichuan. J. Tradit. Chin. Med*. 35,(08): 158-160.
8. Ding, J.R., Zhou, Y.W., Chen, Y. et al. (2012). Effect of Shengmai Injection on cardiac function in elderly patients with coronary heart disease and chronic heart failure. *Intl. J.Geriatr.* 33 (001): 8-10.
9. Wang, L., Zhang, D.L. (2013). Clinical observation of Shuxuening Injection in the treatment of senile coronary heart disease and heart failure. *J Math Med*. 26 (001): 107-108.

Chen, D., Wei, W.Q., Liu, D.M. et al. (2019). Safety evaluation of Yiqi Fumai injection in the treatment of senile coronary heart disease complicated with chronic heart failure. *North. pharmacol.* 16 (12): 154-1552.

1. Miao, W.R., Li, F., Hao, J.Q. (2008).Clinical observation of integrated traditional Chinese and Western medicine in the treatment of senile coronary heart disease and congestive heart failure. *J. Emerg. Tradit. Chin. Med*. 17,(05): 581-5824.
2. Hong, C.X., Yin, X.M. (2009).Clinical observation of integrated traditional Chinese and Western medicine in the treatment of senile coronary heart disease and heart failure. *Anhui. Med. J*. 30 (07): 756-758
3. Yang, Y.Juan., Zhang, Y., Lv. J. et al. (2016). Treatment of 30 cases of senile coronary heart disease complicated with chronic heart failure with Yiqi Fumai injection. S*haanxi. Tradit. Chin. Med*. 37 (10): 1325-1326
4. Lian, Y.J. (2018). Effect analysis of Shengmai injection on coronary heart failure. *World. Latest. Med. Info.* 18(39):155+157.
5. Chen, J.Y., Li. X.H. (1997). Effect of Shenmai Injection on 52 cases of coronary heart disease and congestive heart failure. *J. Zhejiang. Univ. Tradit. Chin. Med*. 21,(04): 12-13.
6. Sun, L.J., Wang. N.D. (2012). Efficacy analysis of Shenmai injection in the treatment of congestive heart failure and coronary heart disease angina pectoris. *China. Pract. Med. J*. 7(26): 164-165.
7. Wang, Y.C., Zhou, L.J., Xiao, Y.L. (2002). 100 cases of coronary heart disease and heart failure treated with Shenmai injection. *J. Hunan. Tradit. Chin. Med*. 18(02):38-39.
8. Xie, Y.M. (1996). Shenmai injection in treating 30 cases of coronary heart failure. *Hunan. J. Tradit. Chin. Med.* 12(2):30.
9. Yang, C.R. (1999). Analysis of 60 cases of cor pulmonale complicated with coronary heart disease and heart failure treated by Shenmai injection. *J. Pract. Med.* 15 (008): 626.
10. Wang, J. (2003). Clinical observation on 66 cases of coronary heart disease and heart failure treated by Xinxuetong. *Tianjin. Pharm.* 15 (001): 24-25.
11. Tian, L.N. (2010). Effect of Shenfu Injection on cardiac function indexes and clinical efficacy in patients with coronary heart disease and heart failure. *Shanxi. Med. J.* 39 (05): 464.
12. Chen, Y., Zhao, Z.L. (2012). Effect of Shenfu Injection on heart rate variability in patients with coronary heart disease and heart failure. *J. Emerg. Tradit. Chin. Med.* 21 (05): 816-817.
13. Liu, L.L., Zhang, H.B. (2020). Effect of Shenfu Injection on heart rate variability in patients with coronary heart disease and heart failure. *J. Math. Med.* 33 (11): 1707-1708.
14. Liang, C.H. (2019).Effects of Shenfu Injection on immune function and cytokines in patients with heart and kidney yang deficiency syndrome of chronic heart failure. *Chron. Pathematol. J.*20 (03): 441-443.
15. Li, S.H., Xie, H.D., Hu, Z.J. et al. (2006).Effect of Shenmai Injection on coronary heart disease and heart failure and heart rate variability. *Continuing. Med. Edu*. 20,(35): 43-44.
16. Wang, Q.X., Liu, C.X. (2004). Effect of Shenmai Injection on plasma cytokine concentration in patients with coronary heart disease and heart failure. P*revent&Treat. Card. Cereb. Dise*.4(04): 52-53 + 55.
17. Xie,W.,Yang,T.L.,Ning,Y.G.(2006).Changes of plasma asymmetric dimethylargine concentration in patients with coronary heart disease and heart failure before and after treatment with Shenmai injection. *Practic. Prevent. Med.* 13(04): 851-853.
18. Zhang, Xiao. (2020).Effect of Xinmailong injection combined with western medicine on LVEDV and LVESV in patients with severe coronary heart disease and heart failure. *China. Disaster. Rescue. Med.* 8 (06): 310-312+315.
19. Yang, X.Y. (2001). Effect of Compound Danshen Injection on calcitonin gene-related peptide and endothelin in patients with coronary heart disease complicated with congestive heart failure. *Chin. J. Integr. Tradit. West. Med.* 21(02): 137.
20. Zhang, X.W. (2020).Effect of Salvia miltiorrhiza polyphenolate injection on coronary heart disease and heart failure and left ventricular function. *Modern. Diagn. Treat.* 31 (10): 1581-1582.
21. Cui, W., Xu, X.Y. (2002).Observation on the efficacy of puerarin injection in the treatment of coronary heart disease and heart failure. *J. Pract. Card*. 11(04): 261.
22. Wang, S.M., Ye,L.F., W, L.H.(2020). Shenmai Injection Improves Energy Metabolism in Patients With Heart Failure: A Randomized Controlled Trial. *Front Pharmacol.* 11(undefined), 459. doi:10.3389/fphar.2020.00459
23. Dai, S.H. (2012).Clinical observation on the effect of Shengmai and Danshen Injection on heart rate variability in patients with coronary heart disease and heart failure. *J Hubei Univ Chin Med.* 32 (08): 13-14.
24. Lu, S., Li, X.Y., Lin, T. et al. (2018).Effect of Shenxiong Glucose Injection on serum Gal-3 and PlGF in patients with chronic heart failure caused by coronary heart disease and its mechanism. *J. Clin. Expe. Med.* 17 (01): 50-54.
25. Ma, C.B., Zhang, J.B., Li, D.Y. (2013).Effect of Shengmai Injection on left ventricular systolic function in patients with coronary heart disease and heart failure by two-dimensional ultrasound speckle tracking. *Jiangsu. Med*.39 (18): 2153-2155.
26. Gong, L,F., Rong, J. (2001).treatment of 120 cases of coronary heart disease and congestive heart failure with integrated traditional Chinese and Western Medicine. *Pract. J. Tradit. Chin. Med*. 17(10): 24.
27. Guo, H.J., Tao, Q.X., Li, S.L. (2012).58 cases of coronary heart disease and chronic heart failure treated with integrated traditional Chinese and Western Medicine. *Jilin. J.Tradit. Chin. Med.*32 (07): 681-683.
28. Chen,Yan., Wang, J.H. (2019).Clinical observation of integrated traditional Chinese and Western medicine in the treatment of coronary heart disease and heart failure. *Pract. J. Tradit. Chin. Med*.35 (02): 206.
29. Wang, X.L., Chen, W.G. (2019).Clinical effect of integrated traditional Chinese and Western Medicine on chronic heart failure of coronary heart disease with Qi deficiency and blood stasis. *Clin. Med. Res. Pract.*4 (02): 143-144.
30. Zhang, L. (2017).clinical observation of integrated traditional Chinese and Western medicine in the treatment of senile coronary heart disease and heart failure. *Elec. J. Card. Cereb. Dise. Int. Trad. Chin. West. Med.* 5 (13): 173.
31. Hong, C.X., Yin, X.M. (2009). Clinical observation of integrated traditional Chinese and Western medicine in the treatment of senile coronary heart disease and heart failure. *Anhui. Med. J.*30 (07): 756-758.
32. Wang, X.L., Zhao, Z.Q., Hou, Y.Z. et al. (2018). Assessment of Complementary Treatment with Yiqi Fumai Lyophilized Injection on Acute Decompensated Ischemic Heart Failure (ACT-ADIHF): Rationale and Design of a Multicenter, Randomized, Controlled Trial. *Cardiovasc. Drugs. Ther.* 32(3), 295-300. doi:10.1007/s10557-018-6791-0
33. Zhao, A.Q. (2016). Study on the clinical effectiveness of combined traditional Chinese and Western medicine in improving the quality of life of patients with coronary heart disease and heart failure. *World. Latest. Med. Info*.16 (41): 146+153.
34. Piao, Z.H. (2015). To explore the effect of integrated traditional Chinese and Western Medicine on chronic heart failure of coronary heart disease. *Elec. J. Card. Cereb. Dise. Int. Trad. Chin. West. Med.*3 (32): 42-43.
35. Jiang, Yin., Shang, H.C. (2018). Clinical evidence and effect mechanism of Yiqi Fumai (freeze-drying) for injection in the treatment of coronary heart disease and heart failure. *World Sci Tech-Modern Tradit Chin. Med*.20 (12): 2141-2144.
36. Luo, Y.M. (2017).Clinical efficacy analysis of Shenfu Injection in the treatment of coronary heart disease and heart failure. *J. Xizang. Med.* 38 (01): 36-38.
37. He, J.Y. (2013).Clinical observation of Breviscapine Injection in the treatment of coronary heart disease and heart failure. *China. Health. Nutrition (zhongxunjian).* (10): 656-657.
38. Wei, H.M., Wu, H.J., Yu.W. et al. (2018). Shenfu decoction as adjuvant therapy for improving quality of life and hepatic dysfunction in patients with symptomatic chronic heart failure. *J Ethnopharmacol*.04(169) :347-55. doi:10.1016/j.jep.2015.04.016
39. Xue, J.G., Xu, Y., Deng, Y.et al.(2019). The Efficacy and Safety of Xinmailong Injection in Patients with Chronic Heart Failure: A Multicenter Randomized Double-Blind Placebo-Controlled Trial. *J. Altern. Complement. Med.* 25(8), 856-860.

doi:10.1089/acm.2019.0030

1. Liang, T., Zhang, Q. (2014).Clinical study of Xinmailong in the treatment of three coronary artery diseases complicated with heart failure. *Henan. Trad. Chin. Med.* (B11): 250-251.
2. Zong, B., Wang, H.B., Zong, X.F. (2014).Clinical study on Shenfu Injection in the treatment of patients with heart failure after emergency PCI for acute myocardial infarction. *Chin. J. Tradit. Chin. Med*. 29 (11): 3640-3642.
3. Wang, Hao. (2013). Clinical observation of Danhong injection in the treatment of patients with acute exacerbation of coronary heart disease and heart failure. *China. Med Eng*. 21 (11): 70.
4. Ju, Y.P., Zhou, C., Lin, S.L. et al. (2008). Clinical efficacy of Danhong injection in the treatment of acute exacerbation of coronary heart disease and heart failure. *China. Pract. Med. J*. 3(35): 133-134.
5. Yan, L.B., Gu, X.R. (2009).Observation on clinical efficacy of Danhong injection in the treatment of heart failure exacerbation of coronary heart disease. *Pract. J. Cardiac. Cereb. Pneum. Vasc. Dis*.17 (07): 579-580.
6. Chen, C.J. (2013).Danhong injection in the treatment of 100 cases of coronary heart disease with cardiac insufficiency. *Tradit. Chin. Med. Clin. Res*.5 (01): 59-60.
7. Zhang,F. (2015). Effect of Shuxuetong Injection on coronary heart disease complicated with heart failure with normal ejection fraction. *Chin. J. Int. Med. Cardio/Cereb. Dis.*13 (03): 356-358.
8. Ge, T.J., Sun, W.X. (1999). Treatment of 30 cases of intractable heart failure with coronary heart disease by Integrated Traditional Chinese and Western Medicine. J Pract Tradit Chin Med. 15(09): 26.
9. Zhu, Y.J., Wang, Q. (2019).Research progress of integrated traditional Chinese and Western medicine in the treatment of coronary heart disease and chronic heart failure. *J. Guangxi. Univ. Tradit. Chin. Med.* 22 (01): 64-67.
10. Ju, A.C., Luo, R.Z., Qin, S.P. et al. (2018).Pharmacological effect and clinical research progress of Yiqi Fumai (freeze-dried) for injection. *Drug. Evalu. Res.* 41 (03): 354-364.
11. Li, P., Wang, T., Fu, S.F. et al. (2016).Comparison of clinical effects of Shenfu and Shenmai injections based on meta-analysis. *Chin. Herb. Med.* 47 (16): 2949-2959.
12. Jiang, H.P. (2007).Observation on the efficacy of Shenmai injection in the treatment of 30 cases of coronary heart disease with heart failure. *Transportation Med.* 21(01): 44+46.
13. Zhu, X.Q. (2015). Observation on the efficacy of Shenfu Injection in the treatment of coronary heart disease and heart failure. *Asia. Pacific. Tradit. Med.* 11 (17): 126-127.
14. Wang, Bin. (2013). Observation on the efficacy of Shenmai injection in 64 patients with coronary heart disease and chronic heart failure with Qi and yin deficiency syndrome. *Tradit. Chin. Med. Clin. Res*.5 (21): 63-64.
15. Wei, J.J., Zhu, M.J., Wang, Y.X. (2020).Systematic evaluation of the efficacy and safety of Xinmailong injection in the treatment of coronary heart disease and heart failure. *Chin. J. Chin. Materia. Medica.* 45 (19): 4756-4765.doi:10.19540/j.cnki.cjcmm.20200302.505
16. Li, N., Zhang, X.Y. (2019). Research progress on TCM treatment of chronic heart failure of coronary heart disease. *World. Latest. Med. Info*. 19(26):97-98.
17. Yang, M.Y., Du, H., Wang, D.D., et al. (2018). Research progress of Traditional Chinese medicine in treating coronary heart failure. J. Emerge. Tradit. Chin. Med. 27(09):1664-1666+1683.
